# Effects of anabolic and catabolic nutrients on woody plant encroachment after long-term experimental fertilization in a South African savanna

**DOI:** 10.1371/journal.pone.0179848

**Published:** 2017-06-29

**Authors:** Anthony J. Mills, Antoni V. Milewski, Dirk Snyman, Jorrie J. Jordaan

**Affiliations:** 1 Department of Soil Science, Stellenbosch University, Matieland, South Africa; 2 Percy FitzPatrick Institute, DST/NRF Centre of Excellence, University of Cape Town, Rondebosch, South Africa; 3 C4 EcoSolutions, Tokai, South Africa; 4 University of Limpopo, Sovenga, South Africa; Tennessee State University, UNITED STATES

## Abstract

The causes of the worldwide problem of encroachment of woody plants into grassy vegetation are elusive. The effects of soil nutrients on competition between herbaceous and woody plants in various landscapes are particularly poorly understood. A long-term experiment of 60 plots in a South African savanna, comprising annual applications of ammonium sulphate (146–1166 kg ha^-1^ yr^-1^) and superphosphate (233–466 kg ha^-1^ yr^-1^) over three decades, and subsequent passive protection over another three decades, during which indigenous trees encroached on different plots to extremely variable degrees, provided an opportunity to investigate relationships between soil properties and woody encroachment. All topsoils were analysed for pH, acidity, EC, water-dispersible clay, Na, Mg, K, Ca, P, S, C, N, NH_4_, NO_3_, B, Mn, Cu and Zn. Applications of ammonium sulphate (AS), but not superphosphate (SP), greatly constrained tree abundance relative to control plots. Differences between control plots and plots that had received maximal AS application were particularly marked (16.3 ± 5.7 versus 1.2 ± 0.8 trees per plot). Soil properties most affected by AS applications included pH (H_2_O) (control to maximal AS application: 6.4 ± 0.1 to 5.1 ± 0.2), pH (KCl) (5.5 ± 0.2 to 4.0 ± 0.1), acidity (0.7 ± 0.1 to 2.6 ± 0.3 cmol kg^-1^), acid saturation (8 ± 2 to 40 ± 5%), Mg (386 ± 25 to 143 ± 15 mg kg^-1^), Ca (1022 ± 180 to 322 ± 14 mg kg^-1^), Mn (314 ± 11 to 118 ± 9 mg kg^-1^), Cu (3.6 ± 0.3 to 2.3 ± 0.2 mg kg^-1^) and Zn (6.6 ± 0.4 to 3.7 ± 0.4 mg kg^-1^). Magnesium, B, Mn and Cu were identified using principal component analysis, boundary line analysis and Kruskal-Wallis rank sum tests as the nutrients most likely to be affecting tree abundance. The ratio Mn/Cu was most related to tree abundance across the experiment, supporting the hypothesis that competition between herbaceous and woody plants depends on the availability of anabolic relative to catabolic nutrients. These findings, based on more than six decades of experimentation, may have global significance for the theoretical understanding of changes in vegetation structure and thus the practical control of invasive woody plants.

## Introduction

The encroachment of woody plants into rangelands is a growing problem worldwide [1, 2, 3]. Long-term control of such changes depends on an understanding of the ultimate reasons for why establishment of trees is constrained in grassland biomes and within the grassy matrix in savanna biomes. Such an understanding of vegetation structure has remained elusive. Although the literature on the treelessness of grasslands has emphasised the adverse effects of drought, fire and frost on woody plants [4, 5, 6, 7], these factors are likely to be mechanisms rather than causes underlying the natural competitive power of grasses and the consequent suppression or exclusion of even those taxa of trees adapted to such adversities [8].

Notwithstanding the effects of factors such as water availability, fire frequency and intensity of herbivory, the availability of certain nutrient elements is likely to be one ultimate cause of the dominance of trees over herbaceous plants in a particular landscape or region [9, 10]. The ways in which various nutrients in soils affect the competition between herbaceous and woody plants remain, however, unclear. A particular conundrum is that many of the world’s grasslands, shrublands and savannas are warm and wet enough to support closed forests [11, 12, 13]. Several authors have proposed that nutrient deficiencies constrain the establishment of trees in these systems [9, 14, 15, 16, 17, 18, 19, 20, 21, 22, 23]. Yet, even the most nutrient-poor savannas and shrublands appear to have sufficient concentrations of nutrients for the establishment of forests [24]. As Bond [24] notes: “The apparent failure of low nutrient stocks to explain the missing forests on nutrient-poor soils emphasises the need for new ideas on how nutrients, alone or in combination with other factors such as fire, influence vegetation structure”. Bond [24] also urges researchers to “…take a fresh look at how nutrients influence vegetation structure”.

A little-known agronomic approach is to use ratios of elements–such as Ca/Mg–to determine the amount of a particular nutrient required to maximise herbaceous or tree crop production [25]. This is because there are synergistic and antagonistic effects among nutrient elements that affect their uptake by roots and their metabolic roles once they enter living cells. The potential effects of ratios of various nutrients on vegetation structure in natural ecosystems have, however, been neglected in the literature. This is accordingly one approach which would offer a ‘fresh look at how nutrients influence vegetation structure’. Recently, Milewski and Mills [8] provided a theoretical framework (hereafter referred to as the Anabolic/Catabolic Theory) for why ratios of anabolic nutrients (e.g. Mn and Mg) to catabolic nutrients (e.g. Cu and Zn) would play a strong role in competition between herbaceous plants and tree seedlings. The theory holds that herbaceous plants are capable of competitively excluding trees on sites where the supply of catabolic nutrients meets the demand created by the photosynthetic potential of the local environment. A recent study in grasslands and woodlands in New South Wales, Australia, corroborated the theory, finding that the soils under grasslands were richer in nutrients, particularly catabolic nutrients, than those under adjacent woodlands [26].

In this paper, we report on the effects of a long-term experiment of 60 plots at the Towoomba Agricultural Development Centre in a South African savanna, namely Springbokvlakte Thornveld [27], on the abundance of trees and a range of soil properties. The treatments entailed various applications of ammonium sulphate (146–1166 kg AS ha^-1^ yr^-1^; Table 1) and superphosphate (233–466 kg SP ha^-1^ yr^-1^; Table 1) as well as harvesting of hay each year for three decades over the period 1949 to 1981, followed by a further three decades of passive protection. During the period of passive protection there was extremely variable woody encroachment across the experiment, with some plots becoming densely wooded and others resisting invasion. The tree species encroaching the most was the frost- and drought-tolerant *Vachellia karroo*, an acacia known as one of the most important woody invaders of grasslands in South Africa [28, 29]. The original design of the Towoomba experiment was based on an attempt to enhance natural grazing by means of fertilization solely with N, S, P and Ca. However, the fertilization treatments have fortuitously and indirectly provided an invaluable opportunity to determine the effects of a range of soil properties on the abundance of woody plants in this savanna. The period of passive protection in the experiment happened to coincide with the first-recorded worldwide wave of woody encroachment in savannas and formerly treeless grasslands [30, 31, 32, 33]. It thus also fortuitously provides an insight into a current global problem.

**Table 1:** Experimental treatments at Towoomba over the period 1949 to 1981.

Treatment	AS	SP	Treatment	AS	SP	Treatment	AS	SP
AS_0_SP_0_	0	0	AS_0_SP_1_	0	233	AS_0_SP_2_	0	466
AS_1_SP_0_	146	0	AS_1_SP_1_	146	233	AS_1_SP_2_	146	466
AS_2_SP_0_	291	0	AS_2_SP_1_	291	233	AS_2_SP_2_	291	466
AS_3_SP_0_	583	0	AS_3_SP_1_	583	233	AS_3_SP_2_	583	466
AS_4_SP_0_	1166	0	AS_4_SP_1_	1166	233	AS_4_SP_2_	1166	466

AS = ammonium sulphate (kg ha^-1^ yr^-1^); SP = superphosphate (kg ha^-1^ yr^-1^)

In summary, the Towoomba experiment comprised two phases, each spanning approximately three decades. The first phase entailed intensive fertilization, harvesting of grass, and protection from livestock. The second phase–during which woody plant encroachment took place across the experiment–entailed protection from livestock. Fire only occurred twice on the site in the six decades of the experiment. Our central question of cause and effect was ‘which soil properties, at the scale of the entire community of plants, are ultimately responsible for the virtual failure of trees to establish in certain plots in the second phase of the Towoomba experiment?’ In order to answer this, we have focussed on various effects on soil composition not originally envisaged in the design of the experiment. The result is an analysis that links soil properties with tree abundances from within an experiment that spanned more than six decades in an African savanna.

## Methods

### Study area and experimental site

**Fig 1:**
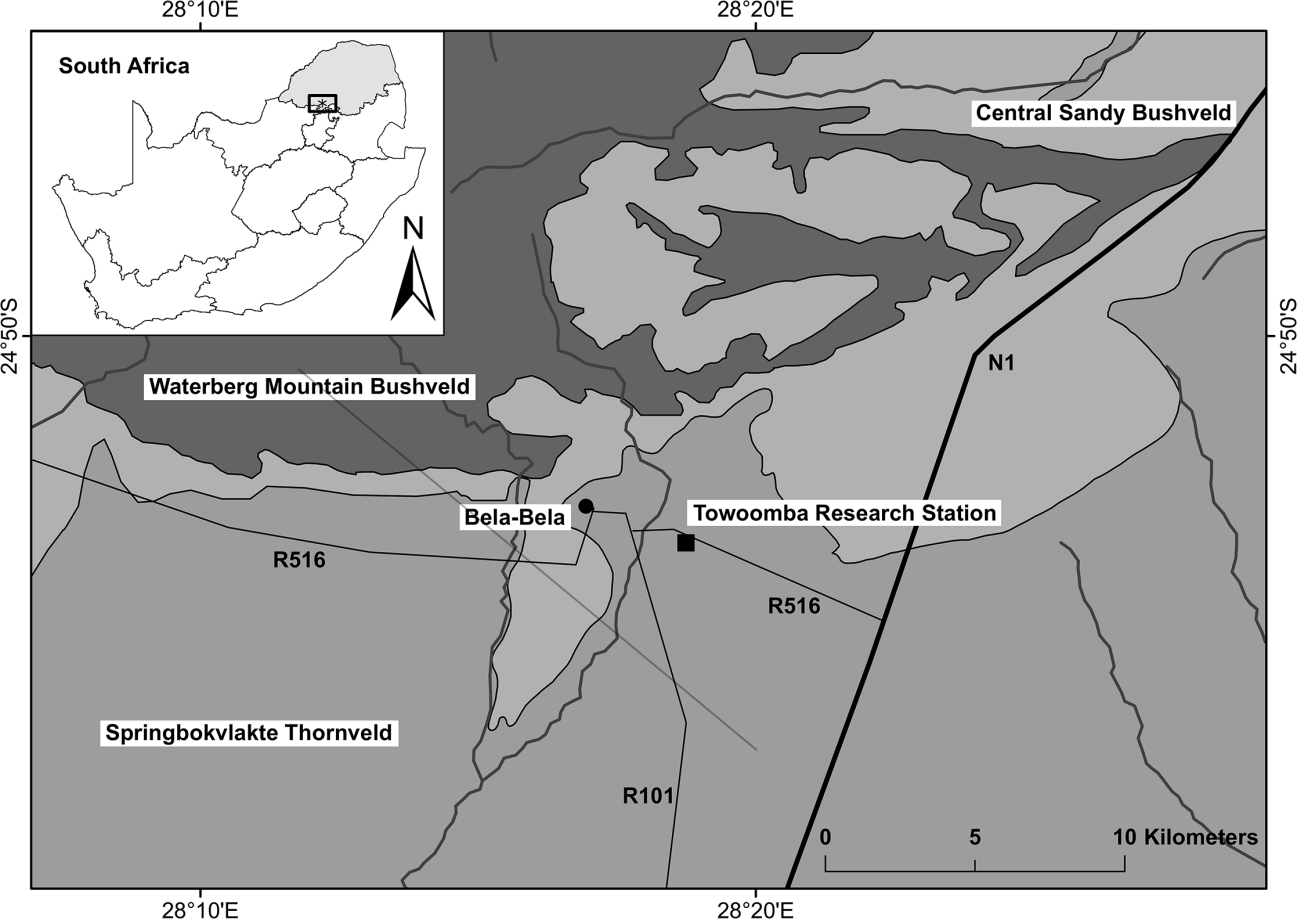
Location of Towoomba Agricultural Development Centre in the southern Springbok Flats of Limpopo Province in South Africa. This map was generated based on datasets from the following sources: [35, 36].

**Fig 2:**
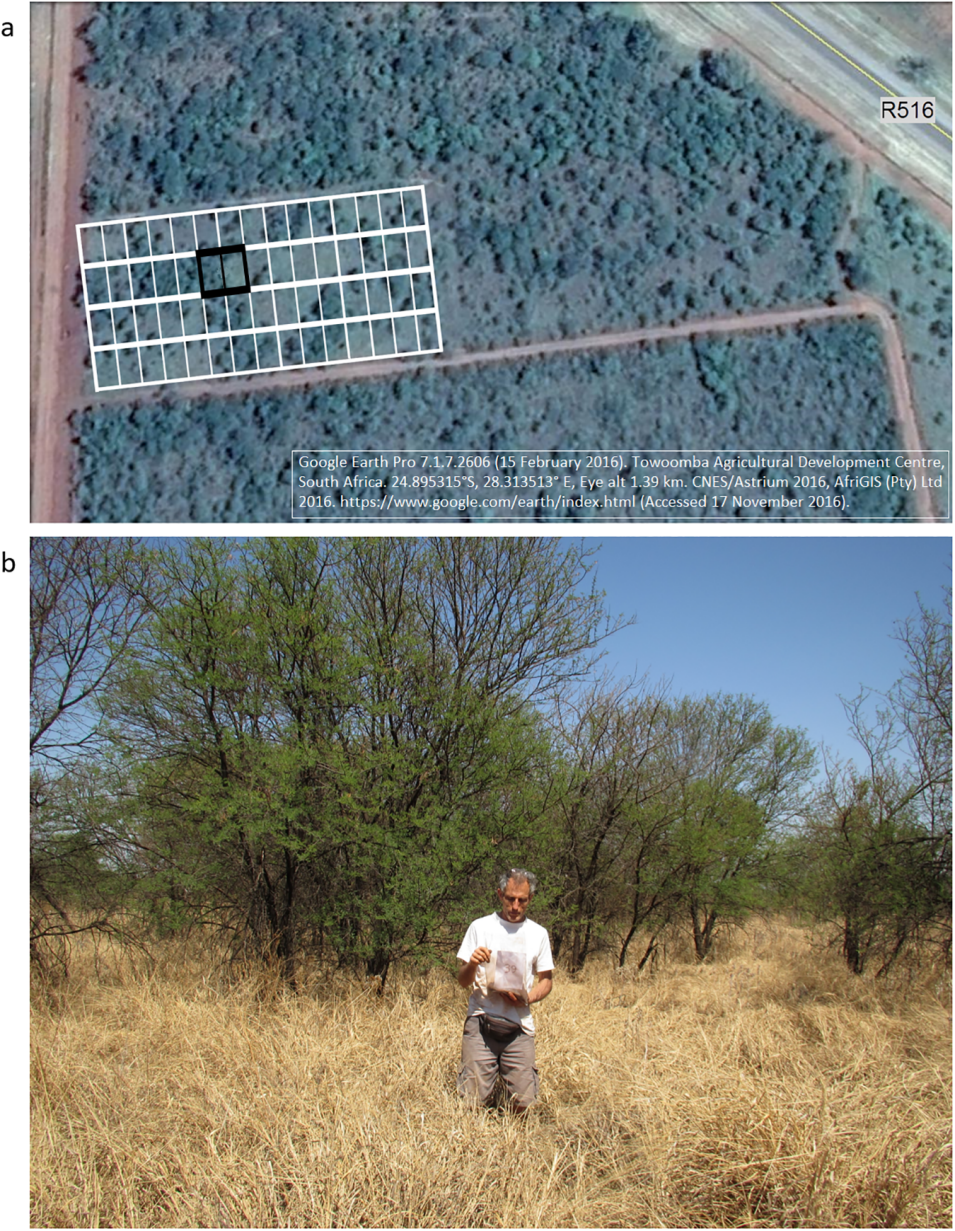
**(a) Satellite image showing the approximate position of the experimental plots at Towoomba Agricultural Development Centre, South Africa; and (b) a photograph taken in September 2014 of two experimental plots.** Black borders in (a) depict the plots shown in (b). Foreground in (b): a plot with no woody encroachment that received the maximum applications of AS (1166 kg ha^-1^ yr^-1^) and SP (466 kg ha^-1^ yr^-1^). Background in (b): a plot with intense woody encroachment by *Vachellia karroo* that received minor applications of AS (146 kg ha^-1^ yr^-1^) and moderate applications of SP (233 kg ha^-1^ yr^-1^). [Depicted is the author Antoni Milewski who confirmed consent for publication].

The Towoomba Agricultural Development Centre (28^o^21’E, 24^o^54’S) is located near Bela-Bela, Limpopo Province, South Africa (Fig 1). This facility was established by the former Transvaal Provincial Administration in 1935. The Towoomba long-term fertilization experiment which we analysed consists of a split-plot design with 15 treatments including controls, totalling 60 plots each measuring 6.4 x 9.1 m [34]. At the outset, a 5 x 3 factorial design was laid out in randomised blocks with four replications–arranged as four rows in the rectangular experimental site (S1 Table). Although the Towoomba experiment has since 1995 been maintained on a reduced budget, the original plots have been preserved and protected to this day (Fig 2).

The study area is representative of the southern Springbok Flats, a plain with soils derived from igneous and sedimentary rocks covered naturally by low open savanna at the time when European farmers arrived around 1870 [27]. The experimental site (120 x 35 m) at Towoomba is level with negligible topography and has freely-drained, base-rich, red-yellow, apedal soil with 30–35% clay in the topsoil, probably derived from dolerites with an alluvial influence [37]. These are rhodic, eutric, haplic ferralsols (corresponding to the Hutton form, Stella family in the South African terminology, [38]. Vertisols (dark cracking clays), derived from basalt [39], occur immediately adjacent to the experimental site but not within the experimental plots.

The study area is mesic with mean annual precipitation (concentrated in summer) of 629 mm and frequent frost, but no snow, in the dry winter. Temperature varies from a mean maximum of 29^°^C and a mean minimum of 16^°^C in December to a mean maximum of 21^°^C and mean minimum of 4^°^C in July [40].

Although the natural savanna in the study area can provide fairly valuable grazing for domestic livestock throughout the year, the rainfall is sufficient in most years to support arable cropping. The rangeland on the Springbok Flats is of mixed value, with some species of grasses remaining attractive as forage for ungulates in the dry winter, while the dormant tissues of others offer only straw insufficient to maintain the body mass of the grazers [27].

Over the last several decades, the remaining unploughed rangelands in the study area have been degraded by widespread encroachment of indigenous woody plants in the absence of any erosion [41]. Given this regional increase in the abundance of trees, a unique opportunity was provided by the fact that the degree of woody encroachment in the experimental site has come to show considerable variation according to the former levels of fertilization among the 60 plots (Fig 2).

The trees and shrubs (hereafter collectively referred to as trees) which have encroached to the greatest extent on the experimental site at Towoomba are *V*. *karroo* and the confamilial *Dichrostachys cinerea*, both of which are capable of symbiotic fixation of N in root nodules [29] (S2 Table). Other plant families contributing to woody encroachment in the study area, but incapable of N-fixation, include e.g. Anacardiaceae (*Searsia lancea*, *S*. *leptodictya*, *S*. *pyroides*), Tiliaceae (*Grewia flavescens*), Combretaceae (*Combretum imberbe*), Ehretiaceae (*Ehretia rigida*) and Ebenaceae (*Diospyros lycioides*).

The common perennial grasses in the experimental site have a tussock form and there is a negligible incidence of lawn-forming species (S1 Text). All grasses have grown spontaneously within the experimental site, with none being deliberately planted. The main species at the start of the experiment–*Themeda triandra*, *Cymbopogon pospischilli* and *Heteropogon contortus*–immediately gave way to others, the sward in certain experimental plots changing temporarily from perennial tussocks to annuals leaving bare ground in the dry season [42]. Fifteen years after the start of the experiment, Louw [42] found the following shifts in the floristic composition of perennial grasses in the plots: with maximal application of AS, *Panicum maximum*, *Urochloa mosambicensis*, *Eragrostis rigidior* and *Brachiaria serrata* increased in abundance; with maximal application of SP, *Hyparrhenia hirta*, *P*. *maximum*, *U*. *mosambicensis* and *Sporobolus stapfianus* increased in abundance; and with maximal application of both AS and SP, there was a 60-fold increase [42] in the abundance of *U*. *mosambicensis*, a favourite food of bovine livestock [43]. Any tendency to woody encroachment during the three decades of treatment was not apparent because of annual cutting of all biomass above a height of 5 cm.

Applications of AS and SP in the experimental site showed toxic effects on several species of grasses [34], but boosted the overall production of hay as follows: the smallest harvests (control treatments) averaged 1.4 t ha^-1^ over three decades and the greatest harvests averaged 5.3 t ha^-1^ by virtue of maximal applications of SP (466 kg ha^-1^) and next-to-maximal applications of AS (583 kg ha^-1^). An average of 5.2 t ha^-1^ of hay was harvested in the treatment with maximal application of both AS and SP (S3 Table). This treatment later experienced the least woody encroachment in the whole experiment. The status of woody plants in the study area over the full course of the Towoomba experiment can be summarised as follows.

1. Before 1949, the incidence of trees in the southern Springbok Flats is unrecorded, but they were probably scarce in what was to become the experimental site.
2. During the period 1949 to 1981, all experimental plots were maintained free of trees by repeated cutting of biomass.
3. During the period 1982 to 1985, some efforts were made to clear trees that started to encroach (mainly by coppicing) on the experimental site, but hay was no longer harvested.
4. During the period 1986 to the present, the experimental site was passively protected with strict limitations on grazing and burning. Only two wildfires (2010 and 2012) occurred. Trees were therefore free to establish and grow among the dense cover of perennial grasses in the experimental plots.

Trees already occurred all around the experimental site in unfertilised savanna by 1985. However, woody encroachment has been so variable that in some plots tree abundance now exceeds that observed in most of the remaining rangeland on the Springbok Flats, whereas in others tree abundance is negligible and thus probably less than that found by the first European farmers around 1870. We assumed that seeds from trees surrounding the experimental site would have dispersed across all plots and that variation in the success of trees in individual plots reflected soil conditions and competition with grass rather than any lack of propagules.

Although the original intention of the Towoomba experiment was simply to fertilise with N, S, P and Ca, the treatments inevitably affected other nutrients via composites, additives and contaminants in the fertiliser formulas, depletion in harvested hay, and changes to availability of certain nutrients by virtue of changes in pH. The only fertilisers ever applied were AS and SP (as per treatments presented in Table 1). The SP fertiliser available for the experimental treatment during the middle of the twentieth century was about two-fold poorer in P than that available today, and was not yet deliberately enriched with Zn. The origin of this original SP would have been rock phosphate mined in Morocco and later locally in South Africa (Phalaborwa and/or Langebaan). At that time, the fertiliser produced from such rock phosphates contained some Mg and impurities including various trace metals [44].

### Sampling and laboratory methods

At the height of the dry season in late September 2014, composite samples of the soil surface–i.e. pedoderm [45]–were taken from all treatment plots to a depth of 2 cm using a glass jar. The pedoderm was sampled because this is the layer of soil in which competition between grasses and tree seedlings is likely to be at its most intense i.e. shortly after germination of the tree seed. Glass was used to prevent any contamination of the soil sample with trace elements from metallic sampling devices. Each composite sample comprised 6–8 subsamples of soil taken randomly from within each plot. Areas under tree crowns were avoided, and any surface leaf litter was removed prior to sampling. Soil samples were air-dried and sieved to <2 mm. Permission to collect soil samples was granted by the Limpopo Department of Agriculture.

A wide range of analyses was undertaken on the composite samples to measure nutrient concentrations and other properties of the soils. pH (H_2_O) and electrical conductivity (EC) were determined using soil/distilled water ratios of 1:5 and 1:2.5, respectively. pH (KCl) was determined in a 1 M KCl solution using a soil/KCl solution ratio of 1:2.5 [46]. Exchangeable acidity was determined using 0.5 mol litre^-1^ potassium sulfate and analysed by means of volumetric titration [46]. Acid saturation was then calculated using the formula: Acid saturation = (Exchangeable acidity ÷ ECEC) × 100; where ECEC = Ca + Mg + K + Na + exchangeable acidity. Water-dispersible clay (WDC) was determined using the method described in Seta and Karathanasis [47]. Inductively Coupled Plasma Mass Spectrometry (ICP-MS) was used to analyse: (i) Na, Mg, K and Ca (extracted in 1% citric acid) [48]; (ii) P (extracted in 1% citric acid [49]; (iii) S (extracted in calcium phosphate [50]; (iv) B (extracted in hot water [51]; and (v) Mn, Zn and Cu (extracted in 0.02M di-ammonium EDTA) [52, 53]. C was determined using the Walkley-Black method [54, 55]. Total N was determined using the Dumas Combustion method [56, 57]. Ammonium and nitrate were extracted using 1 mol dm^-3^ potassium chloride and analysed using the calorimetric method [46].

In terms of toxicity effects in the experiment, we note that long-term application of SP can potentially contaminate soils with heavy metals, particularly As, Cd and Pb [44]. Although we did not analyse for these toxic elements, we note that any adverse effects by As, Cd and Pb would have little explanatory power in terms of woody encroachment because application of SP did not significantly affect the eventual abundance of trees.

The number and height of trees in each experimental plot was recorded between November 2011 and January 2012. We refer throughout this paper to numbers of individual trees per plot as ‘tree abundance’. As previously mentioned, the experimental site has largely been protected from fire despite the cessation of experimental treatments. Only two accidental fires have occurred (during the dry seasons of 2010 and 2012), with no noticeable effects on tree abundance.

### Data analysis

Statistical tests conducted on the data collected included Friedman tests [58], Kruskal-Wallis one-way analyses of variance by ranks [59], Pearson correlation coefficients, principal component analyses (PCA), boundary line analyses, and Kruskal-Wallis rank sum tests. All data were analysed using the statistical software R version 3.3.0 [60]. Non-parametric statistical methods were used for analysing the results of the randomised block design experiment because of the relatively small number of replicates i.e. four plots per treatment. Where Friedman testing showed overall significant differences across treatments (p<0.05), Kruskal-Wallis one-way analyses of variance by ranks were used to investigate whether the AS and/or SP treatments had significant effects on all measured variables. Principal component analyses using R package “ade4” [61] were used to depict in two dimensional space the relationship between tree abundance and all measured soil properties. Boundary line analyses [62, 63] were undertaken on all scatter plots of tree abundance versus soil properties as well as all 91 possible nutrient ratios. The likelihood of a particular boundary line pattern being a result of chance was calculated by running 100,000 iterations of randomised data for each scatter plot (S2 Text). Lastly, Kruskal-Wallis rank sum tests, as well as multiple comparisons run with the R function nparcomp (using Tukey special contrast and a logit transformation), were used to determine which soil properties changed significantly (p<0.05) across four different categories of tree abundance, namely 0–1, 2–4, 4–8 and >8 trees per plot.

## Results

**Fig 3:**
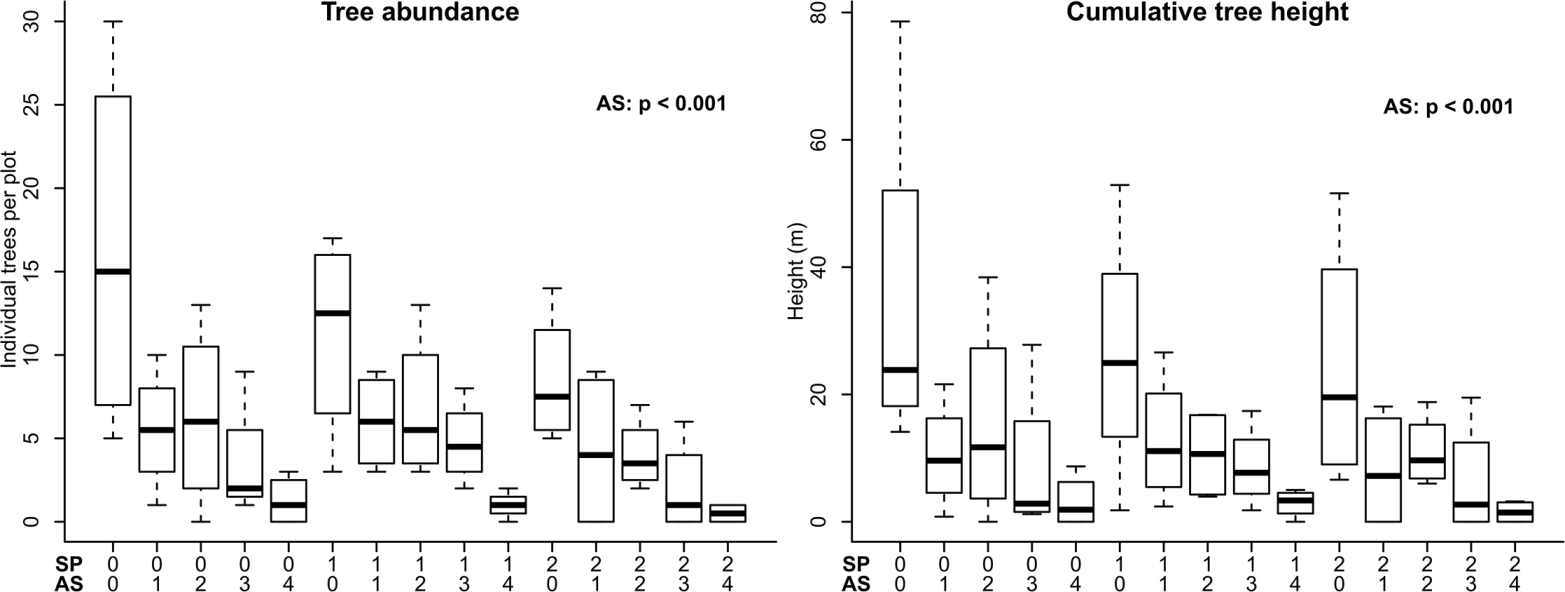
Abundance and cumulative height of trees according to experimental treatment at Towoomba in 2011/12. Box-plots show medians (dark bar), upper and lower quartiles (tops and bottoms of boxes), and ranges (upper and lower whiskers). AS = ammonium sulphate; SP = superphosphate. p values show the results of the Kruskal-Wallis testing for individual effects of AS and SP after Friedman testing for differences across all treatments.

Ammonium sulphate applications over the period 1949 to 1981 had marked effects on subsequent woody encroachment: the greater the fertilization with AS, the more constrained was woody encroachment in terms of both tree abundance (numbers of individuals) and cumulative heights of the same trees (Fig 3 and S5 Table; p<0.001). Control plots had a maximum of 30 and an average of 16.3 (± 5.7) trees per plot compared with a maximum of 3 and an average of 1.3 (± 0.8) trees per plot in plots fertilised with 1166 kg ha^-1^ of AS (Fig 3 and S8 Table). By contrast, fertilization with SP did not have a significant effect on tree abundance (Fig 3 and S6 Table).

Tree abundance was strongly correlated with the cumulative tree height in the same plots (Pearson correlation coefficient of 0.92, S7 Table); of these two variables, tree abundance generally showed the stronger relationships with soil properties and we therefore concentrated on this variable. The woody flora varied little among experimental plots–being dominated by *V*. *karroo* up to 5.3 m high (S2 Table).

**Fig 4:**
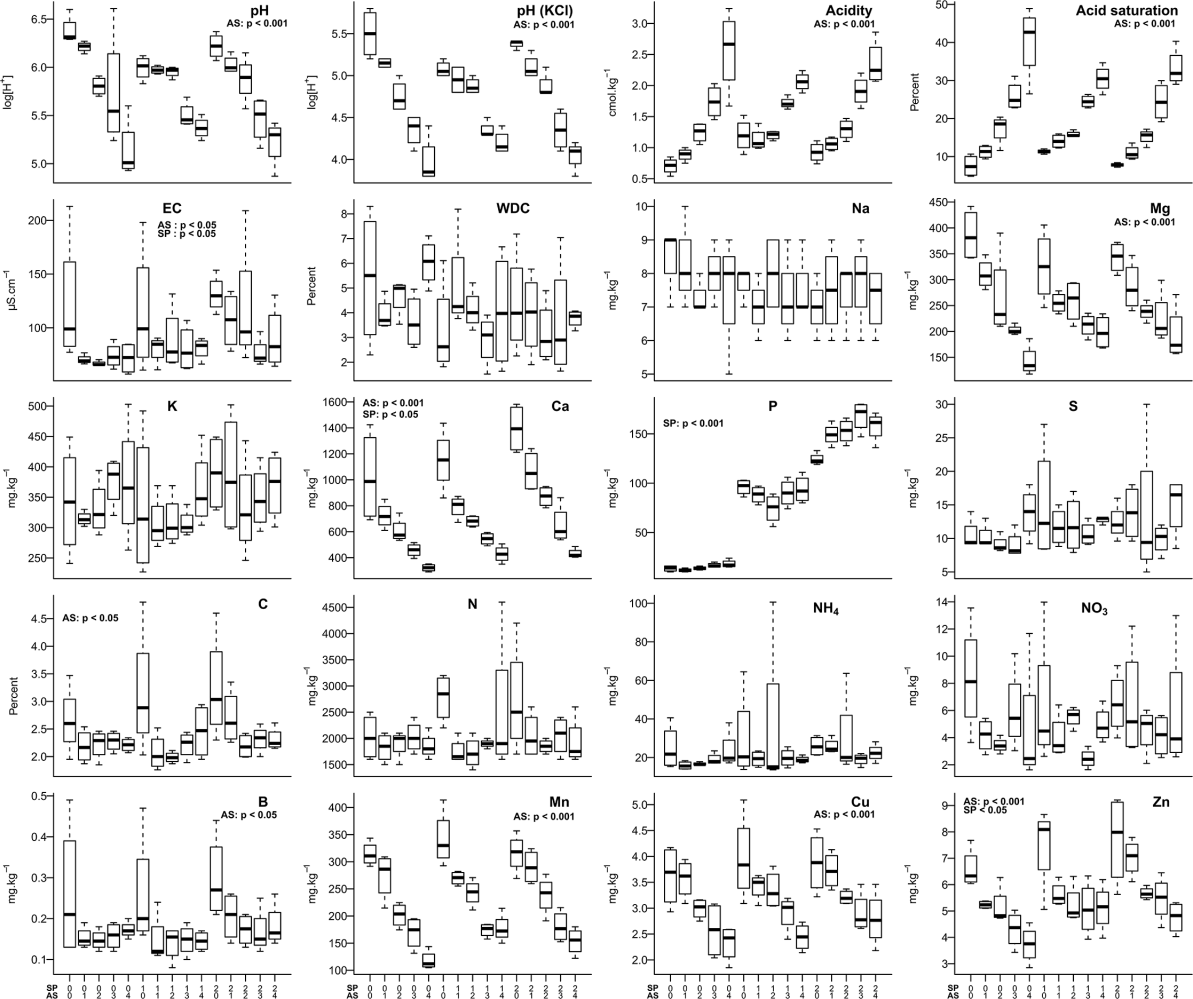
Concentrations of soil properties according to experimental treatment at Towoomba in 2014. Box-plots show medians (dark bar), upper and lower quartiles (tops and bottoms of boxes), and ranges (upper and lower whiskers). AS = ammonium sulphate; SP = superphosphate. p values show the results of the Kruskal-Wallis testing for individual effects of AS and SP after Friedman testing for differences across all treatments.

The results of experimental treatments on soil properties showed statistically significant effects (p<0.05, Friedman and Kruskal-Wallis testing) of AS application on pH, acidity, acid saturation, Mg, Ca, Mn, Cu and Zn (Fig 4; Table 2 and S8 Table). Comparisons between control plots with those receiving the maximal AS application showed a reduction in pH (H_2_O) (6.4 ± 0.1 to 5.1 ± 0.2), a reduction in pH (KCl) (5.5 ± 0.2 to 4.0 ± 0.1), an increase in acidity (0.7 ± 0.1 to 2.6 ± 0.3 cmol kg^-1^; 263% increase), an increase in acid saturation (8 ± 2 to 40 ± 5%; 432%), and depletion of the following nutrients: Mg (386 ± 25 to 143 ± 15 mg kg^-1^; 63% reduction), Ca (1022 ± 180 to 322 ± 14 mg kg^-1^; 69%), Mn (314 ± 11 to 118 ± 9 mg kg^-1^; 62%), Cu (3.6 ± 0.3 to 2.3 ± 0.2 mg kg^-1^; 36%), Zn (6.6 ± 0.4 to 3.7 ± 0.4 mg kg^-1^; 43%), respectively (S9 Table). By contrast, comparisons between control plots with those receiving the maximal SP application showed statistically significant effects on Ca (1022 ± 180 to 1395 ± 95; 36% increase), P (14 ± 1.5 to 124 ± 3.0; 820%) and Zn (6.6 ± 0.4 to 7.7 ± 0.9; 17%) (Table 2 and S9 Table). Notably, there was no effect of AS or SP application on K, S, N, NH_4_, NO_3_ or B.

**Table 2:** Significant effects for ammonium sulphate (AS) and superphosphate (SP) treatments.

	AS statistic	AS p value	SP statistic	SP p value
**pH**	42.8	<0.001	1.08	0.58
**pH (KCl)**	51.3	<0.001	0.37	0.83
**Acidity**	48.1	<0.001	1.17	0.56
**Acid saturation**	52.4	<0.001	0.47	0.79
**Mg**	41.1	<0.001	0.15	0.92
**Ca**	45.6	<0.001	6.63	<0.05
**P**	1.37	0.85	52.5	<0.001
**Mn**	48.9	<0.001	0.94	0.62
**Cu**	36.3	<0.001	2.17	0.34
**Zn**	26.7	<0.001	7.71	<0.05

Test statistics (H values) and p values from non-parametric Kruskal-Wallis one-way analyses of variance by ranks followed by Friedman tests.

**Fig 5:**
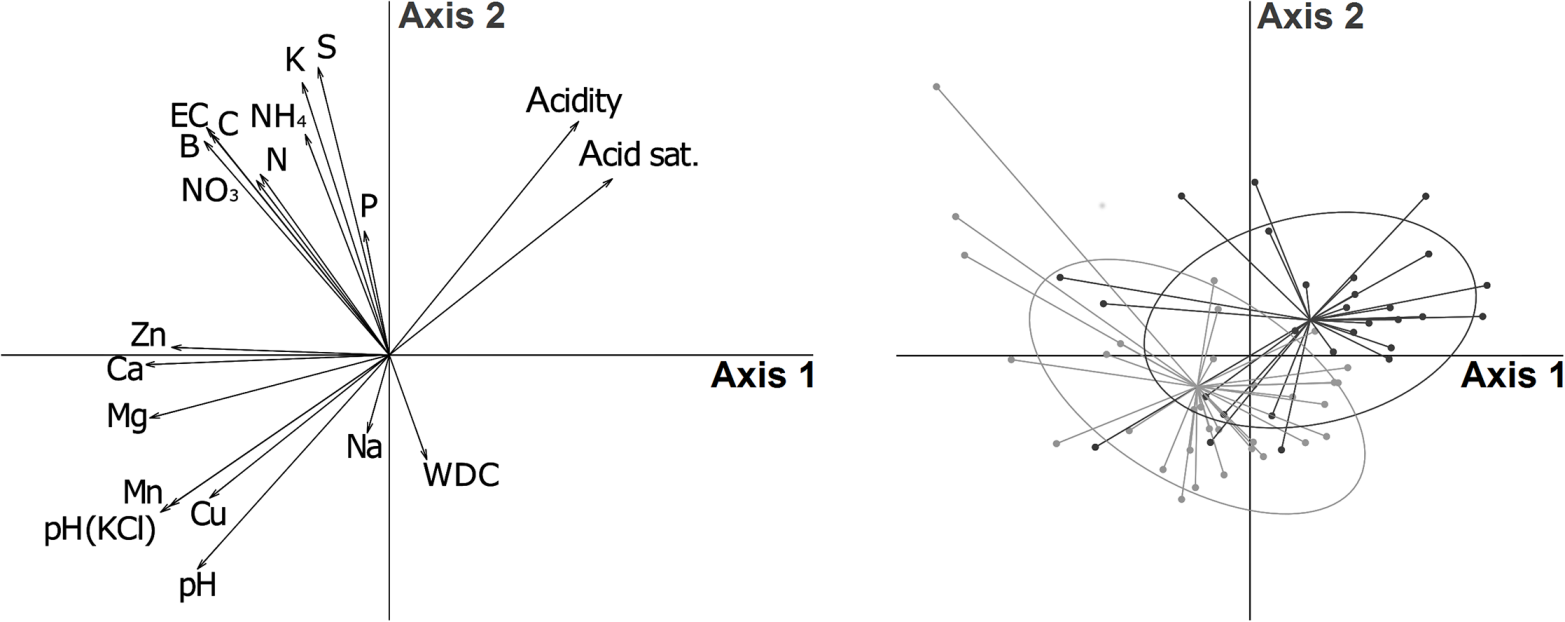
Principal component analysis of tree abundance in relation to all soil properties. Left: arrows indicate the strength of the correlation between each element and the first two axes produced by PCA. Right: points show the scores for the samples on axes 1 and 2 of the PCA, and ellipses summarize the variation in soil samples taken from plots containing 3 or fewer woody plant individuals per plot (black) and those containing 4 or more woody plant individuals per plot (grey).

Soil properties showing strong correlations (i.e. >0.8) included: Mg with Ca (0.84); Mg with Mn (0.84); Ca with Mn (0.80); Ca with Zn (0.81); B with C (0.84); Cu with Mn (0.88); EC with B (0.80); Mn with pH(KCl) (0.84); Ca with pH(KCl) (0.83); Mg with pH(KCl) (0.88); and acid saturation with Mg (-0.82) (S7 Table). In a PCA showing separation of ellipses with tree abundances of 0–3 individual trees per plot from those with four or more (Fig 5), centroids of ellipses were separated from each other with respect to both components in the direction of pH, acidity, acid saturation, Mn and Cu.

**Fig 6:**
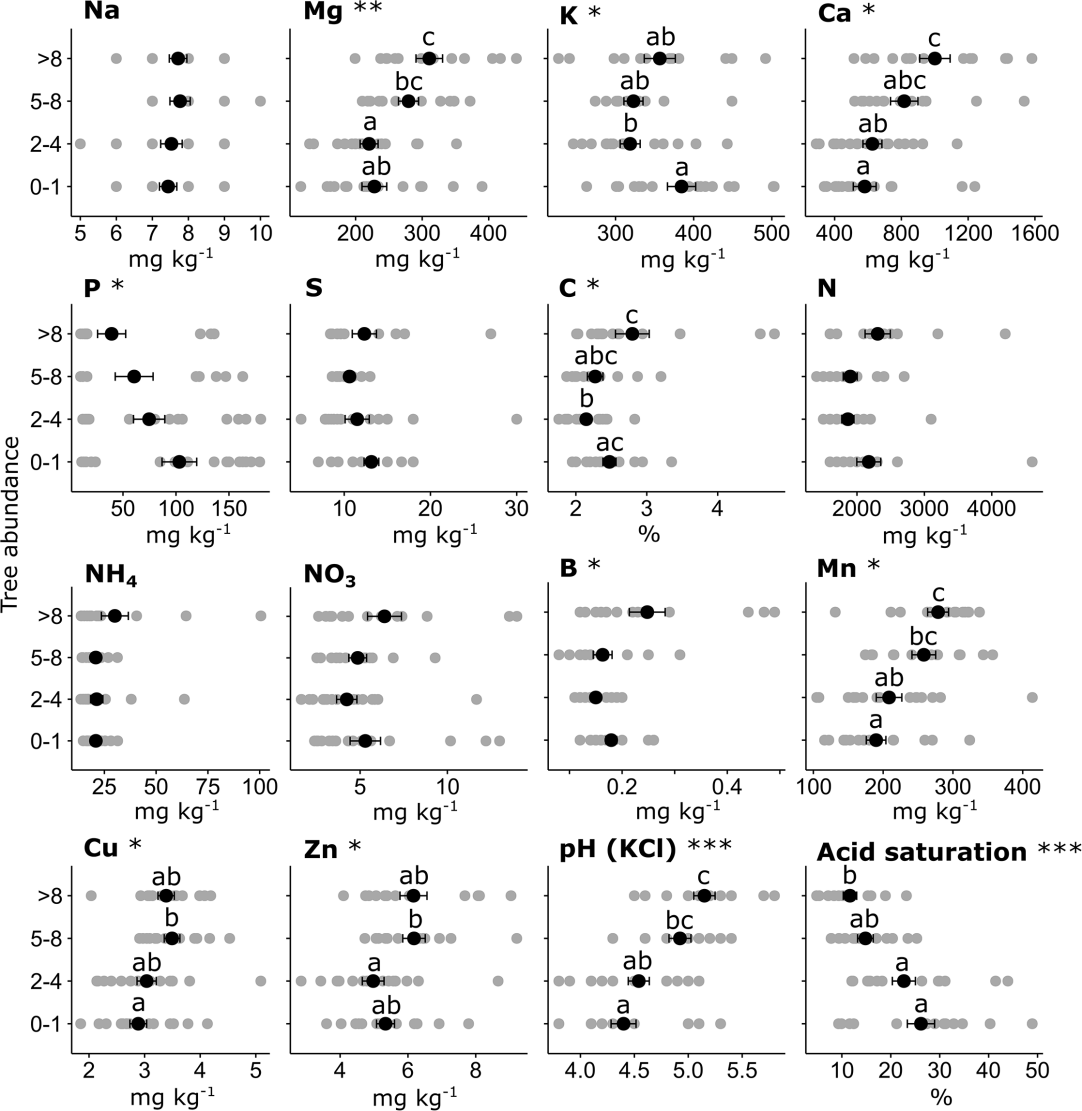
Tree abundance (categorised as 0–1, 2–4, 5–8 or >8 individual trees per plot) relative to nutrient concentrations, pH (KCl) and acid saturation. Data points are depicted as grey circles. Means ± standard errors are depicted with black circles and error bars. Asterisks show significant differences in tree abundance according to Kruskal-Wallis rank sum tests (*** p<0.0001; ** p = 0.001–0.009; * p = 0.01–0.05). Different letters designate significant differences between means (p<0.05).

**Fig 7:**
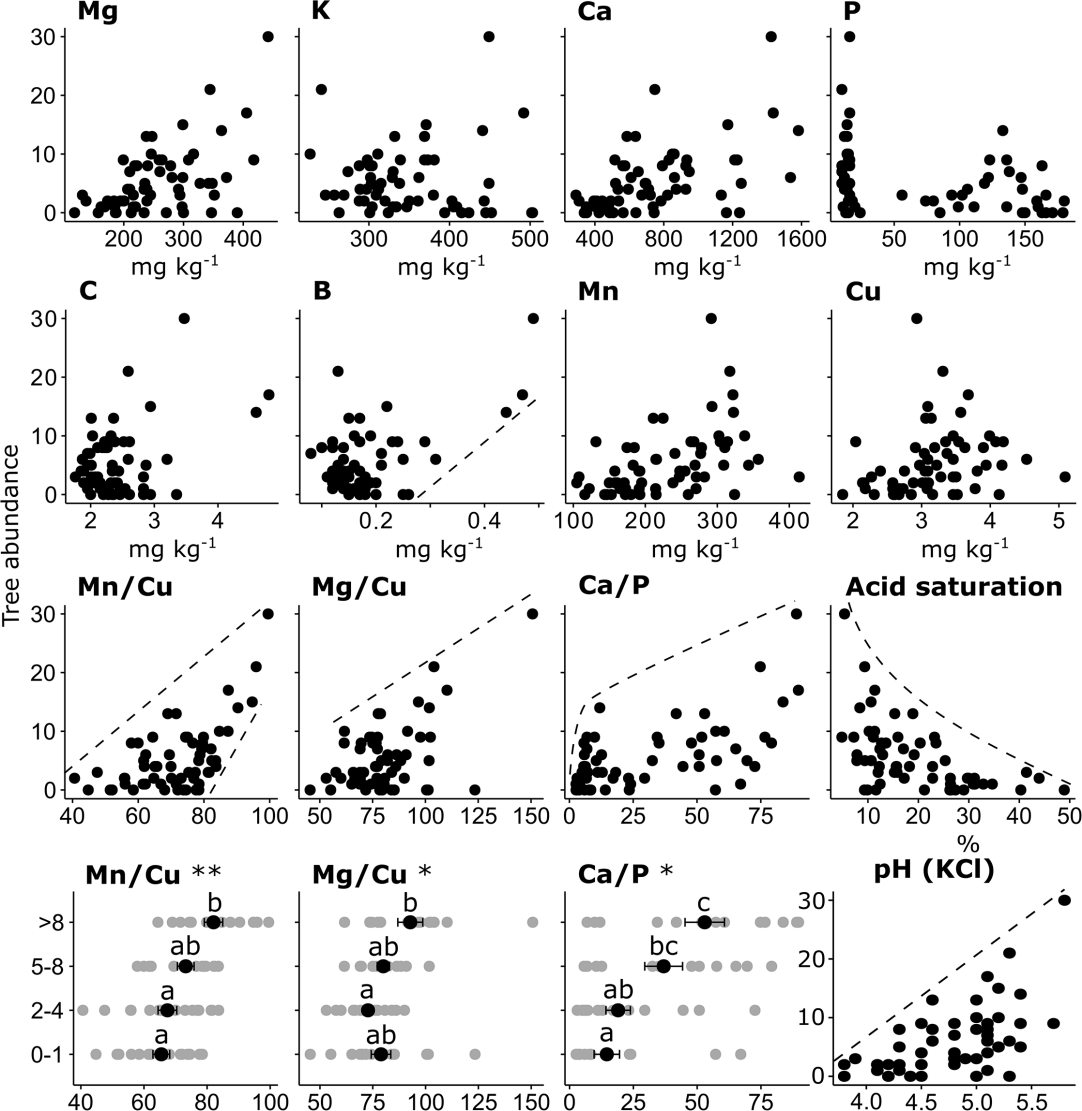
Tree abundance (individual trees per plot) relative to nutrient concentrations, nutrient ratios, pH (KCl) and acid saturation. Dashed lines depict boundary lines identified by boundary line analysis (S4 Table). In the bottom row, four categories of tree abundance (i.e. 0–1, 2–4, 5–8 or >8 individual trees per plot) are shown relative to nutrient ratios, with data points depicted as grey circles, and means ± standard errors depicted with black circles and error bars. Asterisks show significant differences in tree abundance according to Kruskal-Wallis rank sum tests (*** p<0.0001; ** p = 0.001–0.009; * p = 0.01–0.05). Different letters designate significant differences between means (p<0.05).

Soil properties and nutrient ratios that showed strong relationships to tree abundance in both boundary line analyses and Kruskal-Wallis rank sum tests included pH (KCl), soil acidity, acid saturation, B, Mn/Cu, Mg/Cu and Ca/P (Figs 6 and 7 and S3 Fig). Only the ratio Mn/Cu had an upper boundary line depicting a strong constraint on tree abundance with a decrease of the ratio as well as a lower boundary line showing a predictable increase in tree abundance as the ratio increased towards its maximal value.

All raw data used in this study is provided as an online file (S1 Raw Data).

## Discussion

Application of ammonium sulphate (AS) over three decades in the savanna ecosystem of the Springbok Flats greatly constrained woody encroachment over the subsequent three decades. By contrast, application of superphosphate (SP)–despite boosting the concentrations of P in soils by more than an order of magnitude and with the effect still evident three decades after the last application of SP–had no effect on tree abundance (Figs 2 and 3).

Although the main purpose of AS application was to fertilise with N, and although N is regarded as one of the most important nutrients for plants [64, 65], our analyses showed that this element per se had negligible effects on woody encroachment. The soil properties significantly affected by AS application, and therefore possibly constraining tree abundance across the site, were rather pH, soil acidity, acid saturation, Mg, Ca, Mn, Cu and Zn (Fig 4). Such effects of AS on soil properties may be expected because this form of fertiliser is known to acidify soils with concomitant losses of cations via leaching [66, 67]. Furthermore, major reductions in pH, Mg and Ca as a result of AS application in the Towoomba experiment had already been reported by Donaldson et al. [34].

Where acidification was extreme–e.g. plots with pH (KCl) less than 4 and acid saturation greater than 40%–Al toxicity is likely to have affected plant growth. It is conceivable that tree seedlings in the Towoomba experiment were more susceptible than grasses to Al toxicity and that some of the constraint on woody encroachment evident in Figs 6 and 7 is attributable to Al toxicity. However, boundary line analyses (Figs 6 and 7) and Kruskal-Wallis rank sum tests showed marked declines in tree abundance across ranges in pH (KCl) (e.g. 4.5–6) and acid saturation (e.g. 5–25%) in which Al toxicity is implausible. There are therefore likely to be factors beyond acidification that undermined the competitive power of trees at Towoomba.

Boundary line analyses and categorisations of the data according to tree abundance allowed us to distinguish two categories of variables, namely those ensuring constraint of tree abundance and those ensuring promotion of tree abundance. In addition to soil acidity and acid saturation, Ca/P, Mn/Cu and Mg/Cu were associated with constraint, while Mn/Cu and B were associated with promotion. Notably, Mn/Cu was the only variable among all the 20 soil properties measured and the 91 possible nutrient ratios that was reliably associated with both constraint and promotion; it therefore emerged as the variable most strongly related to tree abundance in the experiment. PCA also showed Mn and Cu to be the nutrients most strongly associated with tree abundance (Fig 5).

Although trees are known to enrich soils below their crowns in savannas [68, 69, 70], enrichment of Mg, Ca, P, Mn and Cu by trees in the Towoomba experiment was likely to be minor relative to the effects of the previous applications of AS and SP on these nutrients. This is firstly because concentrations of P and cations had already changed at the end of the fertilization phase of the experiment before any trees had grown [34]. Secondly, enrichment of K, which is characteristic of soils under the crowns of trees [68, 69, 70, 71], was not evident at Towoomba (Fig 7). Indeed, plots with 0–1 trees were more enriched in K than plots with 2–4 trees (Fig 6). An explanation for this is that accumulation of soil organic matter (see graph of C in Fig 6), which strongly adsorbs K [72], was promoted within the dense grass swards of plots with 0–1 trees. And thirdly, a study ~300 km to the southwest of Towoomba–in a similar savanna ecosystem and on soils of similar quality–found that ~7 m tall trees of *Vachellia erioloba*, estimated to be ~35 years old, enriched Ca and Mn only slightly (~ 28 and 8 mg kg^-1^, respectively) and actually impoverished Mg by ~100 mg kg^-1^ [70]. We therefore conclude that, at Towoomba, Mg, Ca, P, Mn and Cu probably affected woody encroachment rather than woody encroachment causing changes in these nutrients. It is particularly notable that N–despite its well-documented importance in natural [73] and agronomic [74] systems–did not show a direct effect on tree abundance in the Towoomba experiment. This is evident in the fact that AS application (Table 1) had no statistically significant effects on total N, NO_3_, and NH_4_ (Table 2 and S8 Table), and that tree abundance was only weakly associated with total N, NO_3_ and NH_4_ relative to other soil properties (Fig 6 and S3 Fig).

The finding of the importance of Mn/Cu supports the Anabolic/Catabolic Theory. Indeed, Milewski and Mills [8] hypothesised as follows: “We predict that where the availability of Cu fails to match that of Mn, a lag arises which leads to the accumulation of photosynthate. Where Cu is insufficient relative to Mn, trees will acquire a competitive advantage over herbaceous plants because the allocation of photosynthate to woody stems is cheap. And conversely, where the availability of Cu is sufficient for the reconstitution of water at the same rate as it is split by Mn, then wood will be expensive, herbaceous plants such as grasses will predominate, and the vegetation will tend to be treeless.”

The Anabolic/Catabolic Theory posits that woody plants differ from herbaceous (non-ligneous) plants in terms of their ratio of demand to supply per unit area for certain nutrient elements indispensable for respiration (i.e. catabolism). Where the supply of catabolic nutrients (e.g. Cu and Zn) is insufficient relative to the demand (as determined by the photosynthetic rate), a surplus of carbohydrate tends to arise [75, 76]. Under these conditions, woody plants are predicted to be competitively superior to herbaceous plants because they capitalise on photosynthetic surpluses by building fibrous structures which eventually augment their supply of scarce nutrients (e.g. deep roots retrieving nutrients from groundwater and large canopies intercepting aerosols). Conversely, where catabolic nutrients are freely available, the specialisation of grasses for deployment of photosynthate in lignin-free tissues means immediate returns in terms of metabolic power and further acquisition of nutrients. This advantage can make grasses competitively superior to trees, by virtue of rapid turnover of nutrients and enhanced capacity to exploit soil resources. In the experimental site at Towoomba, we suggest that this plays out as grasses overwhelming tree seedlings above and below ground and particularly in the pedoderm i.e. the thin layer of topsoil that was sampled in this study. Photographs of plots with minimal tree abundance (Fig 2) show a dense cover of grasses–probably enough to smother tree seedlings even if the soils were otherwise favourable for these seedlings.

Although most nutrients affect various physiological processes in the metabolic cycle and thus form a continuum of overlapping roles rather than mutually exclusive categories [72, 77, 78], some nutrients are generally more involved in anabolic processes than catabolic processes in plants, whereas other nutrients are most involved in enzymes catalysing the breakdown of carbohydrates (see Mills et al. [26]) for additional details of this categorisation). The dichotomy we invoke is plausible because the physiological role of each nutrient, while somewhat versatile, shows signs of our metabolic categorisation. Manganese, for example, is a crucial anabolic catalyst because four of its ions form the centre of the oxygen-evolving complex (OEC), which is the site of water oxidation in Photosystem II (PSII), the first protein complex in photosynthesis [79, 80, 81]. The exact structure of the OEC remains uncertain, but there is little doubt that Mn ions are central to it [77, 82, 83]. By contrast, Cu is an indispensable catalyst catabolically because cytochrome c oxidase–the last enzyme in the respiratory electron transport chain–comprises two Cu centres, CuA and CuB [84]. Indeed, it is arguably the Cu centres that provide the crucial control of electrons in the catalysis of the reconstitution of the water molecule that allows released energy to be efficiently harnessed with minimal release of hazardous free radicals [77, 82, 85, 86, 87]. Because subclinical deficiencies of Cu are (as in the case of B; see below) widespread in agriculture [74], Cu is likely to affect competition between grasses and trees across many parts of the world.

Boundary line analyses and Kruskal-Wallis rank sum tests also identified Mg/Cu, B and Ca/P as being associated with tree abundance in the Towoomba experiment. These variables were subordinate to Mn/Cu in that they were associated with either constraint or promotion, not both. How do they align with the Anabolic/Catabolic Theory; are they mere correlates with Mn/Cu or are they likely to affect tree abundance directly?

Magnesium, like Mn, plays a central role in anabolism because it is indispensable in chlorophyll. The hydrophilic head of the chlorophyll molecule contains a four-pyrrole ring surrounding a central metal ion, namely Mg^2+^. This inner metallic ion cannot be replaced by other ions, even those with similar charges such as Mn^2+^, Zn^2+^, Cd^2+^ or Cu^2+^ [88]. The prominence of the ratio Mg/Cu in the boundary line analysis therefore corroborates the Anabolic/Catabolic Theory.

Boron, like Mn and Mg, is also mainly an anabolic nutrient. The role of B in anabolism of plants is related to its role in stabilising cell membranes and cross-linking of the cell wall component rhamnogalacturonan II. Deficiency of B renders cell walls unstable [89] and hampers their functions, impairing plant growth [90, 91]. There are two reasons why B would greatly affect rates of anabolism and the competitive power of plants in many environments. Firstly, deficiency of B is one of the most important nutritional disorders in agronomy worldwide [92, 93, 94]. And secondly, requirements for B are highly variable among plants. Grasses and their relatives (particularly the order Poales) have minor requirements for B per unit of biomass relative to other monocotyledonous plants and most dicotyledonous plants [95]. The Anabolic/Catabolic Theory would consequently predict that, in many environments, limitation on the supply of B constrains anabolism and therefore the establishment of trees. This prediction was corroborated by the boundary line analysis showing that as B increased in concentration above 0.25 mg kg^-1^ there was a notable increase in tree abundance–evident in a large data-negative zone in the bottom right of the scatter plot.

A positive relationship between growth of trees and concentration of B in soils has been shown previously by means of a nursery experiment on *V*. *karroo*, the commonest species in savanna at Towoomba. In this experiment, the effects of 10 different topsoils from grasslands and savannas in South Africa on seedling growth were investigated [96]. Of all the macro- and micro-nutrients analysed, B was most strongly correlated with growth. The range in concentration of B across these soils (0.16 to 0.46 mg kg^-1^) was smaller than that recorded across the Towoomba experiment (0.08 to 0.49 mg kg^-1^). A strong effect of B on the growth of *V*. *karroo* is therefore unsurprising at Towoomba. Also noteworthy is that B, although unaffected significantly by AS application, varied by nearly an order of magnitude across the experimental site at Towoomba. The relationship between B and tree abundance shown in Figs 6 and 7 thus highlights how woody encroachment was affected by not only AS application but also an intrinsic variation in soil properties across the experimental plots prior to treatment.

The effect of Ca/P on tree abundance in the context of the Anabolic/Catabolic Theory is not as clear as with Mn/Cu and Mg/Cu. Mills et al. [26] note that Ca is mainly an anabolic nutrient and that P is integral to both anabolism and catabolism. It is particularly puzzling that SP application showed no significant effect on tree abundance across the experiment as a whole and yet P showed an association with tree abundance via the ratio Ca/P and in a comparison across different categories of tree abundance (Fig 7). Given the importance of P in natural ecosystems [97] a greater effect of P on woody encroachment at Towoomba might have been expected. The graphs of Ca/P and P do, however, indicate that in the context of the Anabolic/Catabolic Theory P has a greater effect on catabolism than on anabolism. This is because an increase in P was associated with a decrease in tree abundance. The categorisation of P as a mainly catabolic nutrient is unsurprising given that ATP captures the energy released during catabolism of carbohydrates and that a deficiency of P is known to result in considerable accumulation of carbohydrates in both leaves and roots [64, 98].

**Fig 8:**
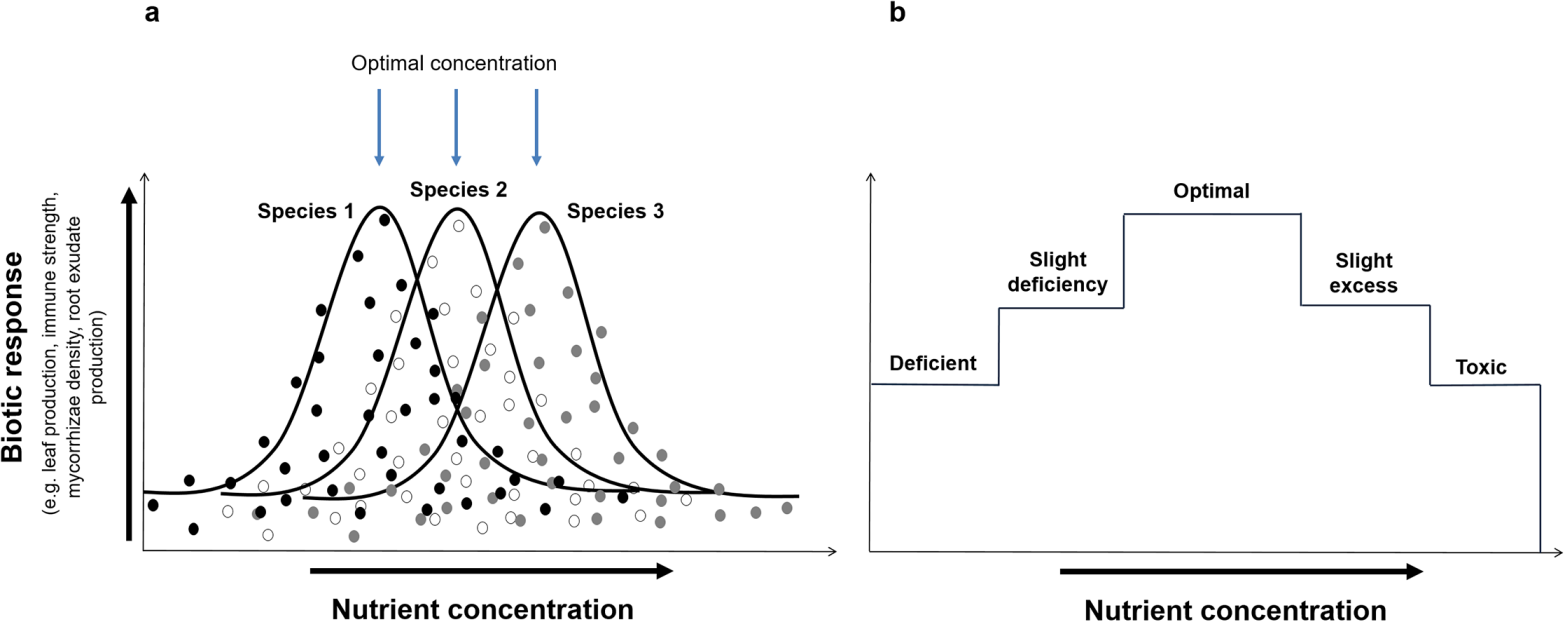
Theoretical relationships between biotic response and nutrient concentration for various plant species occurring in the same environment. The response of plants in terms of vigour to a particular nutrient are invariably bell-shaped curves (a) as opposed to distinct steps (b) delineating zones where concentrations of a particular nutrient are deficient, optimal or toxic.

To summarise the findings of our study in the context of the above-described physiology: the virtual failure of trees to establish in certain plots and not others in the Towoomba experiment can be ascribed mainly to varying availabilities of Mg, Mn, Cu and B, with varying availabilities Ca and P also potentially playing a role. From an agronomic perspective, even the least concentrations of Mg, Ca, P, Mn and Cu measured in the Towoomba experiment would be ample for intensive cultivation of crops. By contrast the concentrations of B would be deemed marginal [74, 99]. A question therefore requiring resolution is: how could Mg, Ca, P, Mn or Cu have affected the establishment of trees if their concentrations at Towoomba were deemed sufficient for agricultural production? A plausible answer is that the competitive power of plants in response to concentrations of a particular nutrient represents a bell-shaped curve [100, 101] as opposed to discrete ranges of values corresponding to deficiency, sufficiency and toxicity Fig 8). The bell-shaped curve in Fig 8 shows that the availability of any particular nutrient will usually be too little or too much for a particular plant; the chances of the concentration being exactly optimal (i.e. maximising the competitive power) are minimal (Fig 8). Consequently, every nutrient is likely to affect–via shortage or excess relative to the optimal concentration–the metabolic power of all plant species present. Furthermore, any constraints exerted are likely to vary markedly among plant species, each of which will have its own particular suite of nutrient requirements in any particular environment (Fig 8). These principles should throw light on how nutrients affect competition between grasses and tree seedlings in the pedoderm.

Sprengel and von Liebig [102, 103] theorised that one or other nutrient will tend to be responsible for constraint in any given agricultural situation, and that unless fertilization with that particular nutrient occurs there will be no increase in crop growth even if fertilization with other nutrients is attempted. Known as the Sprengel-Liebig Law of the Minimum, this has become an accepted principle in agronomy [104, 105]. Valid as it may be, this principle is unlikely to be relevant for competition between grasses and tree seedlings in an ecosystem context such as the Towoomba experiment. This is firstly because coexisting plant species will all have slightly different nutrient requirements, making it unlikely that the same nutrient will be crucial for all plants (Fig 8). Secondly, the competitive power of any species in the plant community reflects a wide range of physiological processes all of which will show different demands for particular nutrients. The capacity of a plant 1) to produce molecules such as allelochemicals and root exudates, and 2) to support populations of mycorrhizae and symbiotic bacteria, will for example determine how effective it is at usurping space and competing with neighbouring plants. Such capacities are strongly affected by nutrition; the availability of each nutrient element will consequently have manifold effects on competition by any particular plant species. Compounding this complexity, the availability of each nutrient varies over time as soil redox, for example, changes in relation to water content, while also varying spatially–often by orders of magnitude over a few centimetres [106]. Other unmeasured complications include: (i) the phenological flux in the demand for nutrients for different plant species; and (ii) many other potentially relevant soil properties (e.g. Mo, Ni, Fe, soil water content, clay type, aggregate stability, soil depth, Al toxicity) and environmental factors (beneficial vs pathogenic microbes, mycorrhizae, intensities of fire vs herbivory). It is beyond the scope of any single study to analyse the complete tangle of correlated factors potentially influencing tree/grass competition over three decades. Fortunately, however, the design of the Towoomba experiment, the boundary line analyses, and Kruskal-Wallis rank sum tests allowed us to tease out the effects of certain salient factors.

In conclusion, determining the precise effects of Mg, Mn, Ca, P and Cu on tree/grass competition at Towoomba would require further research on a wide range of physiological processes. In the interim, the results from this experiment can potentially inform the control of woody encroachment in many rangelands across the world. Although management by means of artificial fertilization of natural ecosystems may seem antithetical to conservation, it is noteworthy that atmospheric deposition of a wide range of nutrients from human activities is already widespread on Earth [107, 108, 109]. An understanding of how manipulation of nutrient ratios affects tree/grass competition could potentially be applied in nutritional approaches to the manipulation of vegetation structure. For example, in attempts to mitigate industrial pollution (e.g. from coal-fired power stations) of atmospheric Mn and Cu, it might help if regional regulations were strict for Mn but lax for Cu. Based on our study, another potentially feasible tactic for controlling woody encroachment would be to fertilise soils directly with Cu in order to boost the competitive power of indigenous grasses. This could be particularly cost-effective for reducing germinative regeneration of trees after poisoning or mechanical clearing. Agronomic experience in fertilizing crops with Cu suggests that as little as 1 kg per hectare could effectively promote the grasses [110]. Rigorous experimentation to determine the efficacy and ecological effects of such fertilization would, however, be required before large-scale implementation could be recommended.

## Supporting information

S1 Table Layout of the Towoomba experiment showing the 5 x 3 factorial design with four replications for each treatment. [See file number 1; “S1 Table.doc”.]
(DOCX)

S2 Table Tree species and their maximum height in 2011/12, according to experimental treatment. Values are numbers of individual trees. Abbreviations in parentheses indicate the species with the maximum height for each plot (V. k. = *Vachellia karroo*; V. ger. = *Vachellia gerrardii*; V. r. = *Vachellia robusta*). AS = ammonium sulphate; SP = superphosphate. [See file number 2; “S2 Table.doc”.]
(DOCX)

S3 Table Mean yields of hay (tonnes ha^-1^) and standard error (SE) in different experimental treatments at Towoomba over the period 1949 to 1981. AS = ammonium sulphate; SP = superphosphate. [See file number 3; “S3 Table.doc”.]
(DOCX)

S4 Table Results of boundary line analyses on soil properties and nutrient ratios. Inter-quartile factors (IQFs) are presented for data-negative zones. All 91 nutrient ratios were analysed, but only those nutrient ratios with IQFs >1 are presented. NA values indicate instances where zones of no-data were not identified (i.e. fewer than four points delineating the boundary between zones). [See file number 4; “S4 Table.doc”.]
(DOCX)

S5 Table Tree abundance, cumulative height of trees, and soil properties in relation to experimental treatments at Towoomba. Means and standard errors are presented. All soil properties are reported in mg kg^-1^ except where indicated. AS = ammonium sulphate. [See file number 5; “S5 Table.doc”.]
(DOCX)

S6 Table Tree abundance, cumulative height of trees, and soil properties in relation to experimental treatments at Towoomba. Means and standard errors are presented. All soil properties are reported in mg kg^-1^ except where indicated. SP = superphosphate. [See file number 6; “S6 Table.doc”.]
(DOCX)

S7 Table Correlation coefficient matrix for data on woody plants and soil properties. [See file number 7; “S7 Table.doc”.]
(DOCX)

S8 Table Test statistics (Q value) and p values for non-parametric Friedman tests for one-way repeated measures analysis of variance by ranks across the Towoomba experiment. [See file number 8; “S8 Table.doc”.]
(DOCX)

S9 Table Tree abundance, cumulative height of trees, and soil properties in relation to all experimental treatments at Towoomba. Means and standard errors are presented. All soil properties are reported in mg kg^-1^ except where indicated. AS = ammonium sulphate; SP = superphosphate. [See file number 9; “S9 Table.doc”.]
(DOCX)

S1 Text Additional information on the vegetation in the Towoomba experiment. [See file number 10; “S1 Text.doc”.]
(DOCX)

S2 Text Boundary line analyses. [See file number 11; “S2 Text.doc”.]
(DOCX)

S1 Fig Theoretical relationship between biotic response and any given soil property. A boundary line separates a data-negative zone from a data-positive zone in a scatter plot [i, ii], which enables delineation of bands of constraint as well as potential for the y variable. [See file number 12; “S1 Fig.tif”.]
(TIF)

S2 Fig Relationship between inter-quartile factor (IQF) and the quartiles of a distribution of random permutations of x and y values. For -1<IQF<0, the observed data (OD) falls between the median and the 1^st^ quartile (Q1) for values smaller than the median, or the 3^rd^ quartile (Q3) for values larger than the median. For 0<IQF<1, OD falls between Q1 and Q1–1.5 times the inter-quartile range (IQR) for values smaller than the median or Q3 and Q3 + 1.5 times IQR for values larger than the median. For IQF >1, OD is greater than Q1–1.5 times IQR for values smaller than the median or Q3 + 1.5 times IQR for values larger than the median. [See file number 13; “S2 Fig.tif”.]
(TIF)

S3 Fig Tree abundance relative to pH (H2O), acidity, electrical conductivity (EC), water-dispersible clay (WDC) and nutrient concentrations. In the top row, four categories of tree abundance (i.e. 0–1, 2–4, 5–8 or >8 individual trees per plot) are shown, with data points depicted as grey circles, and means ± standard errors depicted with black circles and error bars. Asterisks show significant differences in tree abundance according to Kruskal-Wallis rank sum tests (*** p<0.0001; ** p = 0.001–0.009; * p = 0.01–0.05). Different letters designate significant differences between means (p<0.05). [See file number 14; “S3 Fig.tiff”.]
(TIFF)

S1 Raw Data Raw data from which reported results were generated. [See file number 15; “S1 Raw Data.xlsx”.]
(XLSX)
