# Supplementary material for: Effects of anabolic and catabolic nutrients on woody plant encroachment after long-term experimental fertilization in a South African savanna
Source: PLoS One. 2017 Jun 29;12(6):e0179848. doi: 10.1371/journal.pone.0179848 (PMC5491051; doi:10.1371/journal.pone.0179848)
Supplement: S1 Table — [See file number 1; “S1 Table.doc”.] (DOCX) [file pone.0179848.s001.docx]

**S1 Table. Layout of the Towoomba experiment showing the 5 x 3 factorial design with four replications for each treatment.**

|  | 60 | 59 | 58 | 57 | 56 | 55 | 54 | 53 | 52 | 51 | 50 | 49 | 48 | 47 | 46 | Plot number |
| --- | --- | --- | --- | --- | --- | --- | --- | --- | --- | --- | --- | --- | --- | --- | --- | --- |
| H4 | P2 | P1 | P0 | P2 | P0 | P0 | P2 | P0 | P1 | P2 | P2 | P1 | P1 | P1 | P0 | P Treatment |
|  | N4 | N3 | N1 | N3 | N0 | N4 | N2 | N3 | N0 | N1 | N0 | N4 | N2 | N1 | N2 | N Treatment |
|  |  |  |  |  |  |  |  |  |  |  |  |  |  |  |  |  |
|  | 45 | 44 | 43 | 42 | 41 | 40 | 39 | 38 | 37 | 36 | 35 | 34 | 33 | 32 | 31 | Plot number |
| H3 | P0 | P1 | P2 | P2 | P1 | P1 | P2 | P0 | P1 | P0 | P2 | P0 | P2 | P0 | P1 | P Treatment |
|  | N0 | N2 | N2 | N0 | N0 | N1 | N4 | N1 | N4 | N4 | N3 | N3 | N1 | N2 | N3 | N Treatment |
|  |  |  |  |  |  |  |  |  |  |  |  |  |  |  |  |  |
|  | 30 | 29 | 28 | 27 | 26 | 25 | 24 | 23 | 22 | 21 | 20 | 19 | 18 | 17 | 16 | Plot number |
| H2 | P0 | P1 | P2 | P0 | P1 | P2 | P0 | P1 | P0 | P2 | P2 | P2 | P1 | P1 | P0 | P Treatment |
|  | N4 | N1 | N1 | N3 | N4 | N3 | N0 | N2 | N2 | N4 | N0 | N2 | N3 | N0 | N1 | N Treatment |
|  |  |  |  |  |  |  |  |  |  |  |  |  |  |  |  |  |
|  | 15 | 14 | 13 | 12 | 11 | 10 | 9 | 8 | 7 | 6 | 5 | 4 | 3 | 2 | 1 | Plot number |
| H1 | P0 | P1 | P0 | P1 | P2 | P1 | P2 | P2 | P0 | P2 | P1 | P2 | P0 | P1 | P0 | P Treatment |
|  | N3 | N2 | N1 | N3 | N3 | N1 | N2 | N0 | N2 | N1 | N0 | N4 | N4 | N4 | N0 | N Treatment |
