# Supplementary material for: Effects of anabolic and catabolic nutrients on woody plant encroachment after long-term experimental fertilization in a South African savanna
Source: PLoS One. 2017 Jun 29;12(6):e0179848. doi: 10.1371/journal.pone.0179848 (PMC5491051; doi:10.1371/journal.pone.0179848)
Supplement: S2 Table — Values are numbers of individual trees. Abbreviations in parentheses indicate the species with the maximum height for each plot (V. k. = Vachellia karroo; V. ger. = Vachellia gerrardii; V. r. = Vachellia robusta). AS = ammonium sulphate; SP = superphosphate. [See file number 2; “S2 Table.doc”.] (DOCX) [file pone.0179848.s002.docx]

**S2 Table.** **Tree species and their maximum height in 2011/12, according to experimental treatment.** Values are numbers of individual trees. Abbreviations in parentheses indicate the species with the maximum height for each plot (V. k. = *Vachellia karroo*; V. ger. = *Vachellia gerrardii*; V. r. = *Vachellia robusta*). AS = ammonium sulphate; SP = superphosphate.

| **Treatment** | **Tree species** | | | | | | | | | | | **Maximum height (m)** |
| --- | --- | --- | --- | --- | --- | --- | --- | --- | --- | --- | --- | --- |
|  | *Senegalia caffra** | *Senegalia galpinii** | *Vachellia gerrardii** | *Vachellia karroo** | *Vachellia robusta** | *Vachellia tortilis** | *Dichrostachys cinerea** | *Diospyros lycioides* | *Ehretia rigida* | *Grewia flavescens* | *Searsia pyroides* |  |
| AS_0_SP_0_ | 2 |  | 6 | 25 | 6 |  | 8 | 2 | 14 | 1 | 1 | 4.7 (V. k.) |
| AS_1_SP_0_ | 3 |  | 2 | 11 |  |  | 6 |  |  |  |  | 3.1 (V. k.) |
| AS_2_SP_0_ |  |  | 1 | 16 |  |  | 7 |  |  | 1 |  | 4.7 (V. k.) |
| AS_3_SP_0_ |  |  | 5 |  | 5 |  | 4 |  |  |  |  | 3.9 (V. k.) |
| AS_4_SP_0_ | 1 |  | 1 | 1 |  |  | 2 |  |  |  |  | 3.2 (V. ger.) |
| AS_0_SP_1_ | 1 |  | 4 | 17 |  | 3 | 8 |  | 12 |  |  | 4.2 (V. k.) |
| AS_1_SP_1_ | 2 |  | 4 | 9 | 3 |  | 5 | 1 |  |  |  | 3.7 (V. k.) |
| AS_2_SP_1_ | 1 | 1 | 2 | 5 | 4 |  | 8 | 2 | 1 |  |  | 3.1 (V. k.) |
| AS_3_SP_1_ | 1 |  | 2 | 4 | 2 |  | 10 |  |  |  |  | 2.9 (V. r.) |
| AS_4_SP_1_ | 1 |  | 1 | 1 |  |  | 1 |  |  |  |  | 4.1 (V. k.) |
| AS_0_SP_2_ | 2 |  | 6 | 20 | 1 |  | 4 |  | 1 |  | 1 | 4.9 (V. k.) |
| AS_1_SP_2_ |  |  |  | 5 |  | 3 | 8 | 1 |  |  |  | 3.7 (V. k.) |
| AS_2_SP_2_ | 3 |  | 3 | 8 | 2 |  |  |  |  |  |  | 4.2 (V. k.) |
| AS_3_SP_2_ | 1 |  |  | 3 |  |  | 2 |  |  | 2 |  | 5.3 (V. k.) |
| AS_4_SP_2_ |  |  |  | 2 |  |  |  |  |  |  |  | 3.2 (V. k.) |

^*^Nitrogen-fixing species
