# Supplementary material for: Effects of anabolic and catabolic nutrients on woody plant encroachment after long-term experimental fertilization in a South African savanna
Source: PLoS One. 2017 Jun 29;12(6):e0179848. doi: 10.1371/journal.pone.0179848 (PMC5491051; doi:10.1371/journal.pone.0179848)
Supplement: S3 Table — AS = ammonium sulphate; SP = superphosphate. [See file number 3; “S3 Table.doc”.] (DOCX) [file pone.0179848.s003.docx]

**S3** **Table.** **Mean yields of hay (tonnes ha^-1^) and standard error (SE) in different experimental treatments at Towoomba over the period 1949 to 1981.** AS = ammonium sulphate; SP = superphosphate.

| **Treatment** | **Mean hay yield** | **SE** | **Treatment** | **Mean hay yield** | **SE** | **Treatment** | **Mean hay yield** | **SE** |
| --- | --- | --- | --- | --- | --- | --- | --- | --- |
| AS_0_SP_0_ | 1.41 | 0.14 | AS_0_SP_1_ | 1.69 | 0.16 | AS_0_SP_2_ | 1.95 | 0.22 |
| AS_1_SP_0_ | 2.43 | 0.25 | AS_1_SP_1_ | 2.70 | 0.26 | AS_1_SP_2_ | 3.10 | 0.37 |
| AS_2_SP_0_ | 2.58 | 0.29 | AS_2_SP_1_ | 3.64 | 0.40 | AS_2_SP_2_ | 3.34 | 0.36 |
| AS_3_SP_0_ | 2.31 | 0.28 | AS_3_SP_1_ | 4.79 | 0.60 | AS_3_SP_2_ | 5.30 | 0.65 |
| AS_4_SP_0_ | 1.98 | 0.24 | AS_4_SP_1_ | 4.47 | 0.58 | AS_4_SP_2_ | 5.22 | 0.66 |
