# Supplementary material for: Effects of anabolic and catabolic nutrients on woody plant encroachment after long-term experimental fertilization in a South African savanna
Source: PLoS One. 2017 Jun 29;12(6):e0179848. doi: 10.1371/journal.pone.0179848 (PMC5491051; doi:10.1371/journal.pone.0179848)
Supplement: S4 Table — Inter-quartile factors (IQFs) are presented for data-negative zones. All 91 nutrient ratios were analysed, but only those nutrient ratios with IQFs >1 are presented. NA values indicate instances where zones of no-data were not identified (i.e. fewer than four points delineating the boundary between zones). [See file number 4; “S4 Table.doc”.] (DOCX) [file pone.0179848.s004.docx]

**S4 Table. Results of boundary line analyses on soil properties and nutrient ratios.** Inter-quartile factors (IQFs) are presented for data-negative zones. All 91 nutrient ratios were analysed, but only those nutrient ratios with IQFs >1 are presented. NA values indicate instances where zones of no-data were not identified (i.e. fewer than four points delineating the boundary between zones).

|  | **Top left**  **data-negative**  **zone** | **Top right**  **data-negative**  **zone** | **Bottom right**  **data-negative**  **zone** |
| --- | --- | --- | --- |
| **pH (H_2_O)** | 0.6 | NA | 0.8 |
| **pH (KCl)** | 1.0 | NA | 0.2 |
| **Acidity** | NA | 1.0 | NA |
| **Acid saturation** | NA | 1.1 | NA |
| **EC** | -0.1 | NA | NA |
| **WDC** | NA | NA | -0.4 |
| **Na** | -0.3 | 0.1 | NA |
| **Mg** | 0.9 | NA | NA |
| **K** | NA | NA | NA |
| **Ca** | 0.2 | NA | 0.4 |
| **P** | NA | 0.1 | NA |
| **S** | -0.2 | NA | NA |
| **C** | 0.2 | NA | NA |
| **N** | NA | 0.8 | NA |
| **NH_4_** | 0.6 | NA | NA |
| **NO_3_** | 0.6 | NA | NA |
| **B** | 0.1 | NA | 1.6 |
| **Mn** | 0.4 | -0.2 | NA |
| **Cu** | NA | -0.2 | NA |
| **Zn** | 0.1 | 0.5 | NA |
| **Mn/Cu** | 1.2 | NA | 3.6 |
| **Mg/Cu** | 1.2 | NA | NA |
| **Ca/P** | 1.7 | NA | 0.01 |
| **C/N** | 0.9 | NA | 1.5 |
