# Supplementary material for: Effects of anabolic and catabolic nutrients on woody plant encroachment after long-term experimental fertilization in a South African savanna
Source: PLoS One. 2017 Jun 29;12(6):e0179848. doi: 10.1371/journal.pone.0179848 (PMC5491051; doi:10.1371/journal.pone.0179848)
Supplement: S5 Table — Means and standard errors are presented. All soil properties are reported in mg kg-1 except where indicated. AS = ammonium sulphate. [See file number 5; “S5 Table.doc”.] (DOCX) [file pone.0179848.s005.docx]

**S5 Table. Tree abundance, cumulative height of trees, and soil properties in relation to experimental treatments at Towoomba.** Means and standard errors are presented. All soil properties are reported in mg kg^-1^except where indicated. AS = ammonium sulphate.

|  | **AS_0_** | **AS_1_** | **AS_2_** | **AS_3_** | **AS_4_** |
| --- | --- | --- | --- | --- | --- |
| **# trees/plot^1^** | 12.0 ± 2.27 | 5.25 ± 1.05 | 5.67 ± 1.18 | 3.42 ± 0.87 | 0.92 ± 0.29 |
| **Tree height (m)^2^** | 28.5 ± 6.40 | 10.4 ± 2.54 | 12.3 ± 2.95 | 7.85 ± 2.59 | 2.53 ± 0.78 |
| **pH (H_2_O)** | 6.20 ± 0.06 | 6.07 ± 0.04 | 5.88 ± 0.04 | 5.57 ± 0.11 | 5.24 ± 0.07 |
| **pH (KCl)** | 5.32 ± 0.07 | 5.07 ± 0.04 | 4.83 ± 0.04 | 4.35 ± 0.05 | 4.08 ± 0.06 |
| **Acidity (cmol kg^-1^)** | 0.94 ± 0.08 | 1.03 ± 0.05 | 1.25 ± 0.04 | 1.79 ± 0.06 | 2.33 ± 0.13 |
| **Acid saturation (%)** | 8.90 ± 0.69 | 12.1 ± 0.63 | 16.1 ± 0.72 | 24.9 ± 1.01 | 34.6 ± 2.11 |
| **EC (µs cm^-1^)** | 123 ± 13.4 | 85.8 ± 6.46 | 136 ± 44.0 | 77.0 ± 4.29 | 80.7 ± 5.69 |
| **WDC (%)** | 4.35 ± 0.65 | 4.32 ± 0.44 | 3.99 ± 0.31 | 3.39 ± 0.44 | 4.62 ± 0.50 |
| **Na** | 7.75 ± 0.28 | 7.58 ± 0.34 | 7.58 ± 0.26 | 7.67 ± 0.31 | 7.42 ± 0.34 |
| **Mg** | 352 ± 15.7 | 284 ± 11.2 | 254 ± 14.8 | 213 ± 8.88 | 178 ± 12.9 |
| **K** | 357 ± 25.7 | 336 ± 20.1 | 322 ± 15.8 | 344 ± 13.6 | 369 ± 19.8 |
| **Ca** | 1189 ± 84.8 | 861 ± 54.5 | 719 ± 38.9 | 551 ± 34.2 | 394 ± 19.5 |
| **P** | 77.9 ± 14.3 | 83.1 ± 17.1 | 80.3 ± 17.4 | 91.6 ± 18.9 | 89.8 ± 17.45 |
| **S** | 12.6 ± 1.53 | 11.9 ± 0.92 | 11.53 ± 1.9 | 9.86 ± 0.56 | 13.81 ± 0.92 |
| **C** | 3.02 ± 0.26 | 2.32 ± 0.13 | 2.13 ± 0.06 | 2.27 ± 0.06 | 2.33 ± 0.09 |
| **N** | 2508 ± 214 | 1875 ± 93.8 | 1825 ± 67.6 | 1992 ± 75.3 | 2092 ± 243 |
| **NH_4_** | 26.8 ± 4.08 | 20.3 ± 1.54 | 27.6 ± 7.71 | 19.3 ± 0.97 | 21.6 ± 1.74 |
| **NO_3_** | 7.09 ± 1.08 | 4.89 ± 0.78 | 4.5 ± 0.39 | 4.21 ± 0.68 | 5.12 ± 1.05 |
| **B** | 0.27 ± 0.04 | 0.17 ± 0.02 | 0.15 ± 0.01 | 0.16 ± 0.01 | 0.17 ± 0.01 |
| **Mn** | 324 ± 10.8 | 278 ± 8.69 | 228 ± 9.22 | 174 ± 6.73 | 150 ± 9.56 |
| **Cu** | 3.82 ± 0.18 | 3.57 ± 0.09 | 3.18 ± 0.08 | 2.79 ± 0.12 | 2.52 ± 0.12 |
| **Zn** | 7.26 ± 0.40 | 5.96 ± 0.26 | 5.35 ± 0.17 | 4.95 ± 0.27 | 4.53 ± 0.26 |

^1^Cumulative height of all trees per plot
