# Supplementary material for: Effects of anabolic and catabolic nutrients on woody plant encroachment after long-term experimental fertilization in a South African savanna
Source: PLoS One. 2017 Jun 29;12(6):e0179848. doi: 10.1371/journal.pone.0179848 (PMC5491051; doi:10.1371/journal.pone.0179848)
Supplement: S6 Table — Means and standard errors are presented. All soil properties are reported in mg kg-1 except where indicated. SP = superphosphate. [See file number 6; “S6 Table.doc”.] (DOCX) [file pone.0179848.s006.docx]

**S6 Table. Tree abundance, cumulative height of trees, and soil properties in relation to experimental treatments at Towoomba.** Means and standard errors are presented. All soil properties are reported in mg kg^-1^except where indicated. SP = superphosphate.

|  | **SP0** | **SP1** | **SP2** |
| --- | --- | --- | --- |
| **# trees/plot** | 6.55 ± 1.70 | 5.95 ± 1.08 | 3.85 ± 0.90 |
| **Tree height (m)^1^** | 14.6 ± 4.17 | 12.2 ± 2.86 | 10.3 ± 2.83 |
| **pH (H_2_O)** | 5.85 ± 0.12 | 5.76 ± 0.06 | 5.76 ± 0.09 |
| **pH (KCl)** | 4.75 ± 0.13 | 4.69 ± 0.08 | 4.75 ± 0.12 |
| **Acidity (cmol kg^-1^)** | 1.43 ± 0.17 | 1.46 ± 0.09 | 1.51 ± 0.13 |
| **Acid saturation (%)** | 20.4 ± 2.87 | 19.2 ± 1.68 | 18.3 ± 2.24 |
| **EC (µs cm^-1^)** | 108 ± 27.1 | 88.8 ± 7.09 | 105 ± 8.14 |
| **WDC (%)** | 4.73 ± 0.36 | 3.9 ± 0.39 | 3.77 ± 0.35 |
| **Na** | 7.9 ± 0.25 | 7.5 ± 0.21 | 7.4 ± 0.22 |
| **Mg** | 262 ± 21.6 | 250 ± 13.1 | 257 ± 14.6 |
| **K** | 348 ± 14.3 | 325 ± 14.3 | 366 ± 15.5 |
| **Ca** | 627 ± 65.0 | 719 ± 61.7 | 883 ± 80.9 |
| **P** | 14.8 ± 0.76 | 88.5 ± 3 | 150.4 ± 4.14 |
| **S** | 10.5 ± 0.62 | 12.4 ± 0.97 | 12.9 ± 1.24 |
| **C** | 2.31 ± 0.08 | 2.38 ± 0.15 | 2.55 ± 0.14 |
| **N** | 1925 ± 65.6 | 2130 ± 168 | 2120 ± 136 |
| **NH_4_** | 20.0 ± 1.62 | 24.8 ± 4.67 | 24.6 ± 2.30 |
| **NO_3_** | 5.31 ± 0.75 | 4.67 ± 0.59 | 5.51 ± 0.68 |
| **B** | 0.18 ± 0.02 | 0.17 ± 0.02 | 0.21 ± 0.02 |
| **Mn** | 215 ± 17.2 | 241 ± 15.5 | 236 ± 15.6 |
| **Cu** | 3.02 ± 0.15 | 3.22 ± 0.15 | 3.3 ± 0.13 |
| **Zn** | 5.00 ± 0.26 | 5.71 ± 0.29 | 6.12 ± 0.31 |

^1^Cumulative height of all trees per plot
