# Supplementary material for: Effects of anabolic and catabolic nutrients on woody plant encroachment after long-term experimental fertilization in a South African savanna
Source: PLoS One. 2017 Jun 29;12(6):e0179848. doi: 10.1371/journal.pone.0179848 (PMC5491051; doi:10.1371/journal.pone.0179848)
Supplement: S7 Table — [See file number 7; “S7 Table.doc”.] (DOCX) [file pone.0179848.s007.docx]

**S7 Table. Correlation coefficient matrix for data on woody plants and soil properties.**

|  | **Tree**  **#^1^** | **Tree**  **Ht^2^** | **pH** | **pH**  **(KCl)** | **Acidity** | **Acid**  **sat^3^** | **EC** | | **WDC** | | **Na** | | **Mg** | | **K** | | **Ca** | | **P** | | **S** | | **C** | **N** | **NH_4_** | **NO_3_** | **B** | **Mn** | **Cu** | **Zn** | **Mn/Cu** | **Mg/Cu** | **Ca/P** |
| --- | --- | --- | --- | --- | --- | --- | --- | --- | --- | --- | --- | --- | --- | --- | --- | --- | --- | --- | --- | --- | --- | --- | --- | --- | --- | --- | --- | --- | --- | --- | --- | --- | --- |
| **Tree #^1^** | **1** | 0.91 | 0.53 | 0.61 | -0.52 | -0.54 | 0.44 | | -0.19 | | 0.11 | | 0.59 | | 0.01 | | 0.53 | | -0.26 | | 0.03 | | 0.40 | 0.14 | 0.29 | 0.35 | 0.50 | 0.46 | 0.22 | 0.37 | 0.59 | 0.62 | 0.48 |
| **Tree Ht^2^** | 0.91 | **1** | 0.45 | 0.54 | -0.42 | -0.47 | 0.51 | | -0.22 | | 0.14 | | 0.57 | | 0.17 | | 0.58 | | -0.17 | | 0.09 | | 0.53 | 0.28 | 0.27 | 0.38 | 0.66 | 0.40 | 0.19 | 0.33 | 0.51 | 0.61 | 0.40 |
| **pH (H_2_O)** | 0.53 | 0.45 | **1** | 0.92 | -0.92 | -0.91 | 0.24 | | -0.01 | | 0.14 | | 0.75 | | 0.01 | | 0.68 | | -0.19 | | -0.18 | | 0.18 | 0.12 | 0.01 | 0.13 | 0.23 | 0.73 | 0.54 | 0.53 | 0.73 | 0.57 | 0.50 |
| **pH (KCl)** | 0.61 | 0.54 | 0.92 | **1** | -0.94 | -0.96 | 0.46 | | -0.04 | | 0.14 | | 0.86 | | 0.09 | | 0.83 | | -0.09 | | 0.00 | | 0.34 | 0.19 | 0.15 | 0.25 | 0.43 | 0.84 | 0.64 | 0.66 | 0.81 | 0.60 | 0.46 |
| **Acidity** | -0.52 | -0.42 | -0.92 | -0.94 | **1** | 0.96 | -0.25 | | 0.04 | | -0.12 | | -0.73 | | 0.04 | | -0.67 | | 0.15 | | 0.17 | | -0.11 | -0.01 | -0.03 | -0.08 | -0.18 | -0.77 | -0.57 | -0.52 | -0.78 | -0.51 | -0.45 |
| **Acid sat^3^** | -0.54 | -0.47 | -0.91 | -0.96 | 0.96 | **1** | -0.39 | | 0.11 | | -0.12 | | -0.84 | | -0.05 | | -0.81 | | -0.01 | | 0.05 | | -0.30 | -0.18 | -0.10 | -0.21 | -0.34 | -0.85 | -0.67 | -0.67 | -0.81 | -0.56 | -0.36 |
| **EC** | 0.44 | 0.51 | 0.24 | 0.46 | -0.25 | -0.39 | **1** | | -0.20 | | -0.06 | | 0.56 | | 0.62 | | 0.64 | | 0.22 | | 0.70 | | 0.67 | 0.41 | 0.68 | 0.55 | 0.80 | 0.35 | 0.24 | 0.53 | 0.39 | 0.54 | 0.05 |
| **WDC** | -0.19 | -0.22 | -0.01 | -0.04 | 0.04 | 0.11 | -0.20 | | **1** | | -0.07 | | -0.05 | | -0.20 | | -0.12 | | -0.22 | | -0.03 | | -0.26 | -0.28 | -0.12 | -0.12 | -0.20 | 0.04 | 0.12 | -0.14 | -0.11 | -0.22 | 0.16 |
| **Na** | 0.11 | 0.14 | 0.14 | 0.14 | -0.12 | -0.12 | -0.06 | | -0.07 | | **1** | | 0.21 | | 0.01 | | 0.04 | | -0.24 | | -0.18 | | 0.05 | 0.11 | -0.14 | -0.14 | 0.01 | 0.11 | 0.05 | 0.01 | 0.16 | 0.26 | 0.31 |
| **Mg** | 0.59 | 0.57 | 0.75 | 0.86 | -0.73 | -0.84 | 0.56 | | -0.05 | | 0.21 | | **1** | | 0.22 | | 0.84 | | -0.06 | | 0.14 | | 0.58 | 0.41 | 0.18 | 0.50 | 0.64 | 0.84 | 0.72 | 0.74 | 0.71 | 0.69 | 0.48 |
| **K** | 0.01 | 0.17 | 0.01 | 0.09 | 0.04 | -0.05 | 0.62 | | -0.20 | | 0.01 | | 0.22 | | **1** | | 0.24 | | 0.10 | | 0.54 | | 0.58 | 0.47 | 0.40 | 0.40 | 0.59 | -0.09 | -0.21 | 0.21 | 0.11 | 0.52 | -0.03 |
| **Ca** | 0.53 | 0.58 | 0.68 | 0.83 | -0.67 | -0.81 | 0.64 | | -0.12 | | 0.04 | | 0.84 | | 0.24 | | **1** | | 0.24 | | 0.18 | | 0.67 | 0.45 | 0.22 | 0.42 | 0.70 | 0.80 | 0.67 | 0.81 | 0.67 | 0.52 | 0.13 |
| **P** | -0.26 | -0.17 | -0.19 | -0.09 | 0.15 | -0.01 | 0.22 | | -0.22 | | -0.24 | | -0.06 | | 0.10 | | 0.24 | | **1** | | 0.23 | | 0.17 | 0.13 | 0.13 | 0.01 | 0.11 | 0.05 | 0.13 | 0.29 | -0.06 | -0.21 | -0.74 |
| **S** | 0.03 | 0.09 | -0.18 | 0.00 | 0.17 | 0.05 | 0.70 | | -0.03 | | -0.18 | | 0.14 | | 0.54 | | 0.18 | | 0.23 | | **1** | | 0.45 | 0.27 | 0.66 | 0.32 | 0.46 | 0.05 | 0.01 | 0.25 | 0.07 | 0.14 | -0.20 |
| **C** | 0.40 | 0.53 | 0.18 | 0.34 | -0.11 | -0.30 | 0.67 | | -0.26 | | 0.05 | | 0.58 | | 0.58 | | 0.67 | | 0.17 | | 0.45 | | **1** | 0.74 | 0.33 | 0.59 | 0.84 | 0.38 | 0.24 | 0.65 | 0.40 | 0.57 | 0.04 |
| **N** | 0.14 | 0.28 | 0.12 | 0.19 | -0.01 | -0.18 | 0.41 | | -0.28 | | 0.11 | | 0.41 | | 0.47 | | 0.45 | | 0.13 | | 0.27 | | 0.74 | **1** | 0.15 | 0.37 | 0.59 | 0.25 | 0.19 | 0.53 | 0.23 | 0.40 | -0.07 |
| **NH_4_** | 0.29 | 0.27 | 0.01 | 0.15 | -0.03 | -0.10 | 0.68 | | -0.12 | | -0.14 | | 0.18 | | 0.40 | | 0.22 | | 0.13 | | 0.66 | | 0.33 | 0.15 | **1** | 0.36 | 0.41 | 0.08 | 0.06 | 0.17 | 0.07 | 0.17 | -0.10 |
| **NO_3_** | 0.35 | 0.38 | 0.13 | 0.25 | -0.08 | -0.21 | 0.55 | | -0.12 | | -0.14 | | 0.50 | | 0.40 | | 0.42 | | 0.01 | | 0.32 | | 0.59 | 0.37 | 0.36 | **1** | 0.63 | 0.29 | 0.28 | 0.35 | 0.21 | 0.39 | 0.19 |
| **B** | 0.50 | 0.66 | 0.23 | 0.43 | -0.18 | -0.34 | 0.80 | | -0.20 | | 0.01 | | 0.64 | | 0.59 | | 0.70 | | 0.11 | | 0.46 | | 0.84 | 0.59 | 0.41 | 0.63 | **1** | 0.37 | 0.28 | 0.50 | 0.35 | 0.59 | 0.16 |
| **Mn** | 0.46 | 0.40 | 0.73 | 0.84 | -0.77 | -0.85 | 0.35 | | 0.04 | | 0.11 | | 0.84 | | -0.09 | | 0.80 | | 0.05 | | 0.05 | | 0.38 | 0.25 | 0.08 | 0.29 | 0.37 | **1** | 0.88 | 0.76 | 0.79 | 0.33 | 0.27 |
| **Cu** | 0.22 | 0.19 | 0.54 | 0.64 | -0.57 | -0.67 | 0.24 | | 0.12 | | 0.05 | | 0.72 | | -0.21 | | 0.67 | | 0.13 | | 0.01 | | 0.24 | 0.19 | 0.06 | 0.28 | 0.28 | 0.88 | **1** | 0.62 | 0.41 | 0.01 | 0.14 |
| **Zn** | 0.37 | 0.33 | 0.53 | 0.66 | -0.52 | -0.67 | 0.53 | | -0.14 | | 0.01 | | 0.74 | | 0.21 | | 0.81 | | 0.29 | | 0.25 | | 0.65 | 0.53 | 0.17 | 0.35 | 0.50 | 0.76 | 0.62 | **1** | 0.66 | 0.44 | 0.02 |
| **Mn/Cu** | 0.59 | 0.51 | 0.73 | 0.81 | -0.78 | -0.81 | | 0.39 | | -0.11 | | 0.16 | | 0.71 | | 0.11 | | 0.67 | | -0.06 | | 0.07 | 0.40 | 0.23 | 0.07 | 0.21 | 0.35 | 0.79 | 0.41 | 0.66 | **1** | 0.64 | 0.33 |
| **Mg/Cu** | 0.62 | 0.61 | 0.57 | 0.60 | -0.51 | -0.56 | | 0.54 | | -0.22 | | 0.26 | | 0.69 | | 0.52 | | 0.52 | | -0.21 | | 0.14 | 0.57 | 0.40 | 0.17 | 0.39 | 0.59 | 0.33 | 0.01 | 0.44 | 0.64 | **1** | 0.52 |
| **Ca/P** | 0.48 | 0.40 | 0.50 | 0.46 | -0.45 | -0.36 | | 0.05 | | 0.16 | | 0.31 | | 0.48 | | -0.03 | | 0.13 | | -0.74 | | -0.20 | 0.04 | -0.07 | -0.10 | 0.19 | 0.16 | 0.27 | 0.14 | 0.02 | 0.33 | 0.52 | **1** |

^1^ Number of trees per plot

^2^ Cumulative height of all trees per plot

^3^ Acid saturation
