# Supplementary material for: Effects of anabolic and catabolic nutrients on woody plant encroachment after long-term experimental fertilization in a South African savanna
Source: PLoS One. 2017 Jun 29;12(6):e0179848. doi: 10.1371/journal.pone.0179848 (PMC5491051; doi:10.1371/journal.pone.0179848)
Supplement: S8 Table — [See file number 8; “S8 Table.doc”.] (DOCX) [file pone.0179848.s008.docx]

**S8 Table.** **Test statistics (Q value) and p values for non-parametric Friedman tests for one-way repeated measures analysis of variance by ranks across the Towoomba experiment.**

|  | **Test statistic** | **p value** |
| --- | --- | --- |
| **pH (H_2_O)** | 46.3 | < 0.001 |
| **pH (KCl)** | 51.7 | < 0.001 |
| **Acidity** | 50.3 | < 0.001 |
| **Acid saturation** | 52.4 | < 0.001 |
| **EC** | 22.3 | 0.07 |
| **WDC** | 16.7 | 0.28 |
| **Na** | 10.5 | 0.72 |
| **Mg** | 42.4 | < 0.001 |
| **K** | 11.9 | 0.61 |
| **Ca** | 50.9 | < 0.001 |
| **P** | 53.3 | < 0.001 |
| **S** | 14.3 | 0.43 |
| **C** | 17.2 | 0.25 |
| **N** | 14.3 | 0.43 |
| **NH_4_** | 19.9 | 0.13 |
| **NO_3_** | 18.1 | 0.20 |
| **B** | 18.4 | 0.19 |
| **Mn** | 52.3 | < 0.001 |
| **Cu** | 43.8 | < 0.001 |
| **Zn** | 35.1 | < 0.005 |
