# Supplementary material for: Effects of anabolic and catabolic nutrients on woody plant encroachment after long-term experimental fertilization in a South African savanna
Source: PLoS One. 2017 Jun 29;12(6):e0179848. doi: 10.1371/journal.pone.0179848 (PMC5491051; doi:10.1371/journal.pone.0179848)
Supplement: S9 Table — Means and standard errors are presented. All soil properties are reported in mg kg-1 except where indicated. AS = ammonium sulphate; SP = superphosphate. [See file number 9; “S9 Table.doc”.] (DOCX) [file pone.0179848.s009.docx]

**S9 Table. Tree abundance, cumulative height of trees, and soil properties in relation to all experimental treatments at Towoomba.** Means and standard errors are presented. All soil properties are reported in mg kg^-1^except where indicated. AS = ammonium sulphate; SP = superphosphate.

|  | **SP_0_AS_0_** | **SP_0_AS_1_** | **SP_0_AS_2_** | **SP_0_AS_3_** | **SP_0_AS_4_** | **SP_1_AS_0_** | **SP_1_AS_1_** | **SP_1_AS_2_** | **SP_1_AS_3_** | **SP_1_AS_4_** | **SP_2_AS_0_** | **SP_2_AS_1_** | **SP_2_AS_2_** | **SP_2_AS_3_** | **SP_2_AS_4_** |
| --- | --- | --- | --- | --- | --- | --- | --- | --- | --- | --- | --- | --- | --- | --- | --- |
| **# trees/plot** | 16.3 ± 5.70 | 5.50 ± 1.85 | 6.25 ± 2.78 | 3.50 ± 1.85 | 1.25 ± 0.75 | 11.3 ± 3.12 | 6.00 ± 1.47 | 6.75 ± 2.25 | 4.75 ± 1.25 | 1.00 ± 0.41 | 8.50 ± 2.02 | 4.25 ± 2.46 | 4.00 ± 1.08 | 2.00 ± 1.41 | 0.5 ± 0.29 |
| **Tree height (m)^1^** | 35.1 ± 14.7 | 10.4 ± 4.30 | 15.6 ± 8.33 | 8.68 ± 6.40 | 3.13 ± 2.06 | 26.2 ± 10.5 | 12.8 ± 5.15 | 10.5 ± 3.60 | 8.65 ± 3.24 | 2.93 ± 1.09 | 24.3 ± 10.2 | 8.13 ± 4.75 | 11.0 ± 2.86 | 6.23 ± 4.60 | 1.53 ± 0.88 |
| **pH (H2O)** | 6.38 ± 0.07 | 6.21 ± 0.03 | 5.81 ± 0.05 | 5.74 ± 0.30 | 5.14 ± 0.16 | 6.00 ± 0.06 | 5.97 ± 0.02 | 5.95 ± 0.03 | 5.50 ± 0.06 | 5.37 ± 0.06 | 6.22 ± 0.07 | 6.03 ± 0.05 | 5.88 ± 0.12 | 5.46 ± 0.12 | 5.22 ± 0.12 |
| **pH (KCl)** | 5.50 ± 0.15 | 5.15 ± 0.03 | 4.75 ± 0.10 | 4.35 ± 0.10 | 3.98 ± 0.14 | 5.08 ± 0.05 | 4.95 ± 0.09 | 4.88 ± 0.05 | 4.35 ± 0.05 | 4.20 ± 0.07 | 5.38 ± 0.03 | 5.10 ± 0.07 | 4.88 ± 0.08 | 4.35 ± 0.12 | 4.05 ± 0.09 |
| **Acidity (cmol kg^-1^)** | 0.71 ± 0.07 | 0.89 ± 0.05 | 1.24 ± 0.08 | 1.74 ± 0.13 | 2.56 ± 0.33 | 1.20 ± 0.13 | 1.13 ± 0.09 | 1.20 ± 0.03 | 1.72 ± 0.05 | 2.06 ± 0.08 | 0.93 ± 0.08 | 1.06 ± 0.05 | 1.30 ± 0.08 | 1.91 ± 0.12 | 2.36 ± 0.18 |
| **Acid saturation (%)** | 7.56 ± 1.45 | 11.2 ± 0.85 | 17.3 ± 1.95 | 25.9 ± 1.92 | 40.2 ± 4.83 | 11.3 ± 0.29 | 14.0 ± 0.92 | 15.9 ± 0.41 | 24.5 ± 0.81 | 30.5 ± 1.74 | 7.81 ± 0.24 | 11.0 ± 0.94 | 15.2 ± 1.02 | 24.4 ± 2.54 | 33.3 ± 2.48 |
| **EC (µs cm^-1^)** | 122 ± 31.1 | 70.4 ± 2.21 | 201 ± 134 | 73.9 ± 5.84 | 71.6 ± 7.38 | 114 ± 30.0 | 80.2 ± 6.57 | 88.4 ± 15.0 | 80.4 ± 10.8 | 80.8 ± 5.15 | 131 ± 8.57 | 107 ± 13.3 | 118 ± 30.7 | 76.6 ± 6.75 | 89.9 ± 14.8 |
| **WDC (%)** | 5.40 ± 1.39 | 3.93 ± 0.33 | 4.66 ± 0.38 | 3.64 ± 0.55 | 6.04 ± 0.47 | 3.29 ± 0.97 | 5.11 ± 1.03 | 4.13 ± 0.40 | 2.91 ± 0.51 | 4.06 ± 1.20 | 4.35 ± 1.04 | 3.93 ± 0.83 | 3.17 ± 0.63 | 3.62 ± 1.21 | 3.76 ± 0.18 |
| **Na** | 8.50 ± 0.50 | 8.25 ± 0.63 | 7.25 ± 0.25 | 8.00 ± 0.41 | 7.50 ± 0.87 | 7.75 ± 0.25 | 7.00 ± 0.41 | 8.00 ± 0.58 | 7.25 ± 0.63 | 7.50 ± 0.50 | 7.00 ± 0.41 | 7.50 ± 0.65 | 7.50 ± 0.50 | 7.75 ± 0.63 | 7.25 ± 0.48 |
| **Mg** | 386 ± 25.4 | 311 ± 14.4 | 266 ± 42.0 | 203 ± 4.68 | 143 ± 15.0 | 326 ± 34.3 | 255 ± 9.89 | 258 ± 20.7 | 212 ± 11.1 | 199 ± 16.6 | 343 ± 15.0 | 287 ± 23.7 | 239 ± 9.06 | 224 ± 25.3 | 194 ± 26.5 |
| **K** | 345 ± 45.4 | 315 ± 5.84 | 331 ± 22.8 | 376 ± 20.3 | 374 ± 49.6 | 337 ± 60.3 | 307 ± 21.7 | 310 ± 20.9 | 307 ± 10.9 | 363 ± 32.0 | 390 ± 32.2 | 387 ± 51.2 | 333 ± 41.0 | 349 ± 26.1 | 369 ± 28.0 |
| **Ca** | 1022 ± 180 | 724 ± 50.0 | 607 ± 47.0 | 458 ± 26.0 | 322 ± 14.1 | 1151 ± 118 | 792 ± 43.1 | 681 ± 21.4 | 545 ± 23.6 | 428 ± 32.6 | 1395 ± 95.0 | 1067 ± 80.0 | 871 ± 39.9 | 651 ± 73.6 | 432 ± 18.6 |
| **P** | 13.5 ± 1.50 | 11.8 ± 0.85 | 13.8 ± 0.85 | 16.8 ± 1.18 | 18.3 ± 2.02 | 96.0 ± 4.02 | 88.3 ± 4.4 | 74.3 ± 7.39 | 90.0 ± 6.98 | 93.8 ± 7.02 | 124 ± 3.04 | 149 ± 5.53 | 153 ± 6.16 | 168 ± 7.69 | 158 ± 7.53 |
| **S** | 10.5 ± 1.17 | 10.2 ± 0.93 | 9.10 ± 0.64 | 9.03 ± 1.01 | 13.8 ± 1.85 | 15.0 ± 4.39 | 11.7 ± 1.41 | 12.03 ± 2.11 | 10.7 ± 0.88 | 12.8 ± 0.25 | 12.4 ± 1.33 | 13.8 ± 2.07 | 13.5 ± 5.62 | 9.90 ± 1.08 | 14.9 ± 2.24 |
| **C** | 2.66 ± 0.31 | 2.19 ± 0.15 | 2.22 ± 0.13 | 2.28 ± 0.09 | 2.21 ± 0.06 | 3.15 ± 0.59 | 2.07 ± 0.17 | 1.99 ± 0.05 | 2.21 ± 0.12 | 2.46 ± 0.25 | 3.24 ± 0.49 | 2.71 ± 0.25 | 2.19 ± 0.11 | 2.32 ± 0.12 | 2.31 ± 0.10 |
| **N** | 2025 ± 221 | 1825 ± 138 | 1900 ± 135 | 2025 ± 149 | 1850 ± 126 | 2775 ± 232 | 1750 ± 119 | 1725 ± 149 | 1900 ± 41.0 | 2500 ± 704 | 2725 ± 533 | 2050 ± 218 | 1850 ± 64.0 | 2050 ± 185 | 1925 ± 229 |
| **N.NH4** | 24.9 ± 5.84 | 15.9 ± 1.06 | 16.6 ± 0.41 | 19.1 ± 1.50 | 23.6 ± 4.82 | 29.7 ± 11.7 | 19.3 ± 2.04 | 36.1 ± 21.5 | 19.8 ± 2.37 | 18.9 ± 0.87 | 25.9 ± 2.60 | 25.8 ± 1.95 | 30.1 ± 11.2 | 19.0 ± 1.54 | 22.4 ± 2.29 |
| **N.NO3** | 8.36 ± 2.05 | 4.17 ± 0.61 | 3.44 ± 0.28 | 6.02 ± 1.50 | 4.56 ± 2.38 | 6.39 ± 2.57 | 4.03 ± 0.82 | 5.52 ± 0.38 | 2.45 ± 0.36 | 4.94 ± 0.65 | 6.53 ± 1.13 | 6.45 ± 2.09 | 4.55 ± 0.85 | 4.15 ± 0.78 | 5.85 ± 2.42 |
| **B** | 0.26 ± 0.09 | 0.15 ± 0.01 | 0.15 ± 0.01 | 0.16 ± 0.02 | 0.17 ± 0.01 | 0.26 ± 0.07 | 0.15 ± 0.03 | 0.14 ± 0.02 | 0.15 ± 0.02 | 0.15 ± 0.01 | 0.30 ± 0.05 | 0.21 ± 0.03 | 0.17 ± 0.02 | 0.17 ± 0.03 | 0.18 ± 0.03 |
| **Mn** | 314 ± 11.1 | 274 ± 21.5 | 202 ± 11.3 | 169 ± 15.0 | 118 ± 8.83 | 342 ± 25.9 | 270 ± 6.29 | 243 ± 12.3 | 174 ± 6.31 | 177 ± 13.4 | 316 ± 18.1 | 290 ± 15.8 | 238 ± 17.8 | 180 ± 14.5 | 153 ± 12.5 |
| **Cu** | 3.62 ± 0.30 | 3.57 ± 0.19 | 2.99 ± 0.10 | 2.57 ± 0.27 | 2.32 ± 0.17 | 3.96 ± 0.42 | 3.42 ± 0.13 | 3.35 ± 0.19 | 2.91 ± 0.17 | 2.44 ± 0.13 | 3.88 ± 0.30 | 3.73 ± 0.18 | 3.21 ± 0.07 | 2.91 ± 0.19 | 2.79 ± 0.26 |
| **Zn** | 6.60 ± 0.37 | 5.24 ± 0.07 | 5.16 ± 0.37 | 4.30 ± 0.35 | 3.73 ± 0.35 | 7.48 ± 0.82 | 5.62 ± 0.23 | 5.23 ± 0.37 | 5.08 ± 0.51 | 5.12 ± 0.45 | 7.70 ± 0.86 | 7.02 ± 0.35 | 5.67 ± 0.12 | 5.47 ± 0.43 | 4.75 ± 0.30 |

^1^Cumulative height of all trees per plot
