# Supplementary material for: Effects of anabolic and catabolic nutrients on woody plant encroachment after long-term experimental fertilization in a South African savanna
Source: PLoS One. 2017 Jun 29;12(6):e0179848. doi: 10.1371/journal.pone.0179848 (PMC5491051; doi:10.1371/journal.pone.0179848)
Supplement: S1 Text — [See file number 10; “S1 Text.doc”.] (DOCX) [file pone.0179848.s010.docx]

**S1 Text. Additional information on the vegetation in the Towoomba experiment.**

The 60 experimental plots in the Towoomba experiment’s randomised block design were subjected to fertilization and annual harvesting of hay (to 5 cm above ground) for more than three decades over the period 1949 to 1981. The hay consisted variously of *Bothriochloa insculpta*, *Cymbopogon plurinodis*, *Elionurus argenteus*, *Heteropogon contortus*, *Hyparrhenia hirta*, *Hyperthelia dissoluta*, *Themeda triandra*, *Aristida* (2 spp.), *Stipagrostis uniplumis*, *Brachiaria* (3 spp.), *Digitaria* (2 spp.), *Panicum* (2 spp.), *Rhynchelytrum repens*, *Urochloa mosambicensis*, *Eragrostis* (4 spp.), *Trichoneura grandiglumis*, *Chloris virgata*, *Cynodon dactylon*, *Microchloa caffra*, *Sporobolus stapfianus*, *Tragus racemosus*, *Enneapogon scoparius* and *Schmidtia pappophoroides*. The experimental site was then preserved by protecting it from all but occasional wildfire and light grazing, leaving the vegetation to further natural change while maintaining the infrastructure of roads, paths, fences, posts and labels. The only recorded infrastructural damage during the entire six decades of the experiment was when parts of two experimental plots at a western corner of the site were slightly disturbed by widening of the adjacent dirt road in 2013. The experimental site thus lay with no further systematic treatment for more than three decades until we sampled the soil surface and photographed all 60 plots in 2014. At that time, all grasses were seasonally dormant but intact owing to the absence of grazers. Although grass-eating species of termites were noted in the experimental plots, we saw no evidence that any insects had been eating the dry grass.
