# Supplementary material for: Effects of anabolic and catabolic nutrients on woody plant encroachment after long-term experimental fertilization in a South African savanna
Source: PLoS One. 2017 Jun 29;12(6):e0179848. doi: 10.1371/journal.pone.0179848 (PMC5491051; doi:10.1371/journal.pone.0179848)
Supplement: S2 Text — [See file number 11; “S2 Text.doc”.] (DOCX) [file pone.0179848.s011.docx]

**S2 Text. Boundary line analyses.**

The use of boundary lines to identify potential effects of environmental factors on biotic responses (S1 Fig) was, as far as we know, first conceptualised by Beaufils [i]. It has since become familiar in agronomy [ii] and ecology (see e.g. iii, iv, v, vi, vii]. By defining boundary lines that separate data-negative zones from data-positive zones within scatter plots of x and y variables from a large dataset [i, ii], bands of constraint [viii] as opposed to bands of potential for the y variable can be graphically depicted (S1 Fig). The bands of constraint delineate areas in the graph where the independent variable x possibly exerts constraint on the dependent variable y. The qualifier ‘possibly’ is important because causality cannot be established but only surmised; the constraint could be from another variable that is correlated with the independent variable. Identification of constraint may nevertheless help to reveal which environmental factors ultimately affect trees. In contrast to the band or bands of constraint, the band of potential delineates an area in the graph where the independent variable appears to exert minimal constraint on the dependent variable. Note however that within this band of potential the dependent variable will usually be constrained by other factors, and consequently only a small proportion of the data points reflect minimal constraint and occur near the boundary line.


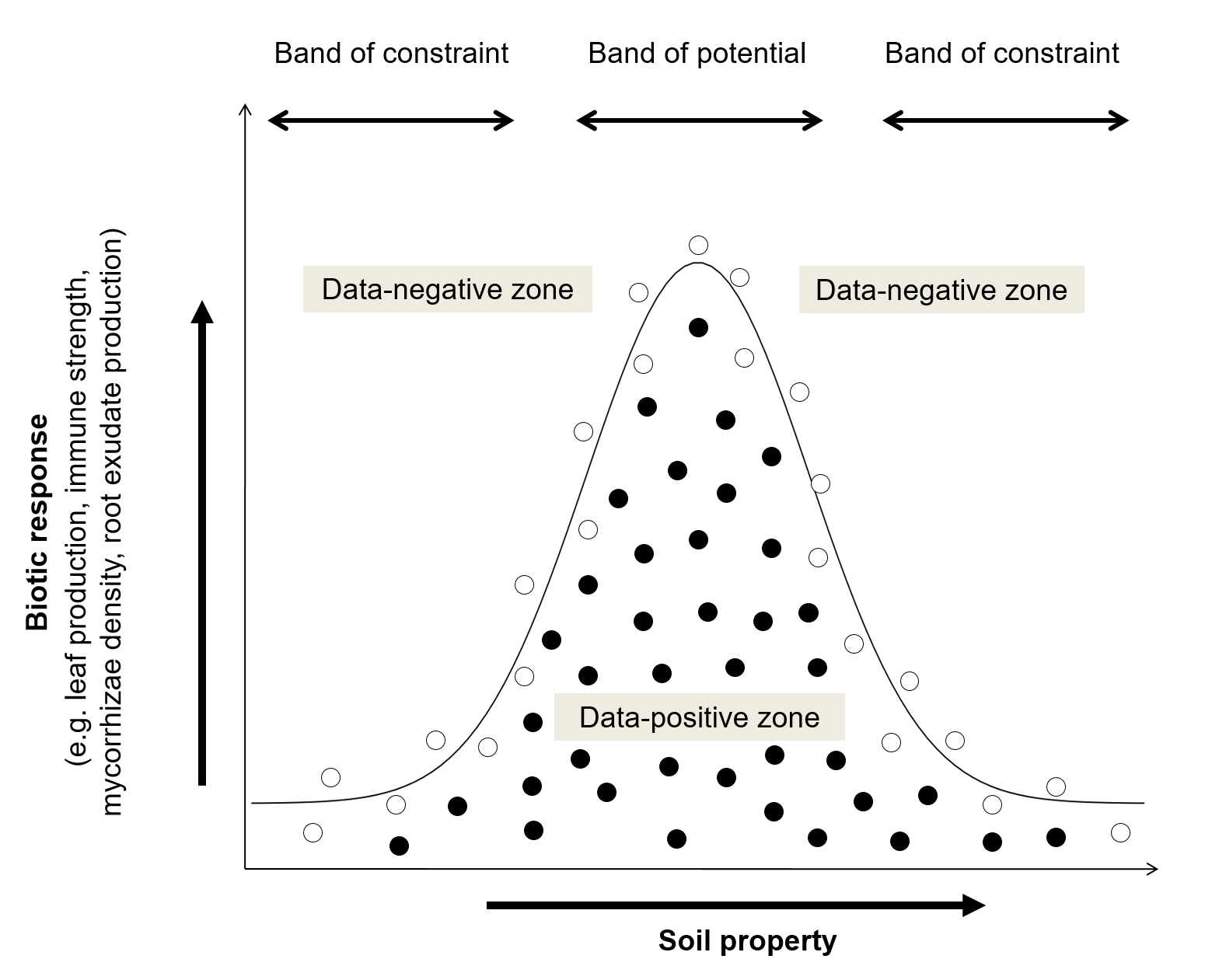


**S1 Fig. Theoretical relationship between biotic response and any given soil property.** A boundary line separates a data-negative zone from a data-positive zone in a scatter plot, which enables delineation of bands of constraint as well as potential for the y variable [i, ii].

Methods previously used to define boundary lines include mathematical models [viii], partitioned regressions [ix], isolation of data points [iii, x], and quantile regression [v]. To identify the soil properties most likely to represent causal factors in our dataset we quantified the likelihood of the data-negative zones being accidental using a random permutation approach. This was done as follows. Boundary points and data-negative zones were identified using the method of Mills et al. [iii]. Areas were calculated for those data-negative zones delineated by at least four boundary points. These areas were then compared with areas obtained from random permutations of the x and y values to determine the likelihood that the observed pattern was the result of chance. As a conservative measure, the point representing maximal x value (i.e. maximum value of the soil property in question) with its corresponding y value as well as the point representing maximal y value (i.e. maximum number of trees) with its corresponding x value were not included in the random permutations, but instead held constant. All other x and y values were randomly resampled to create new x and y value combinations. Boundary points for this new set of x and y values were determined and new areas of data-negative zones for these combinations were calculated. This process was repeated 100,000 times. Box plots were then used to assess to what degree the area of the observed data-negative zone (OD) was an outlier compared with the set of areas of data-negative zones generated through the random permutations (RPD). This comparison was made using what we term an inter-quartile factor (IQF), calculated as follows:

$$For OD>Median \left( RPD \right): \frac{OD-3rd Quartile}{1.5 \times Inter-Quartile Range}$$

$$For OD<Median \left( RPD \right): \frac{OD-1st Quartile}{1.5 \times Inter-Quartile Range}$$

Thus, where IQF is less than zero, the OD falls within the box on the boxplot; where the IQF is between zero and one, the OD falls outside of the box, but inside the whisker; and where IQF is greater than one, the OD falls outside of the whisker (S2 Fig). The whiskers of a boxplot correspond to μ ± 2.698σ in a normally-distributed population (i.e. 99.3% of the population), and are frequently used to identify outliers in a dataset [xi]. We therefore used boxplot whiskers as a means of identifying those soil properties in our dataset which had ODs highly unlikely to result from chance alone. We also used the quantum of all IQF scores exceeding unity, as a means of identifying those soil properties likely to be most affecting woody encroachment. This is because the greater the IQF score above unity, the greater is the likelihood that OD was not accidental.


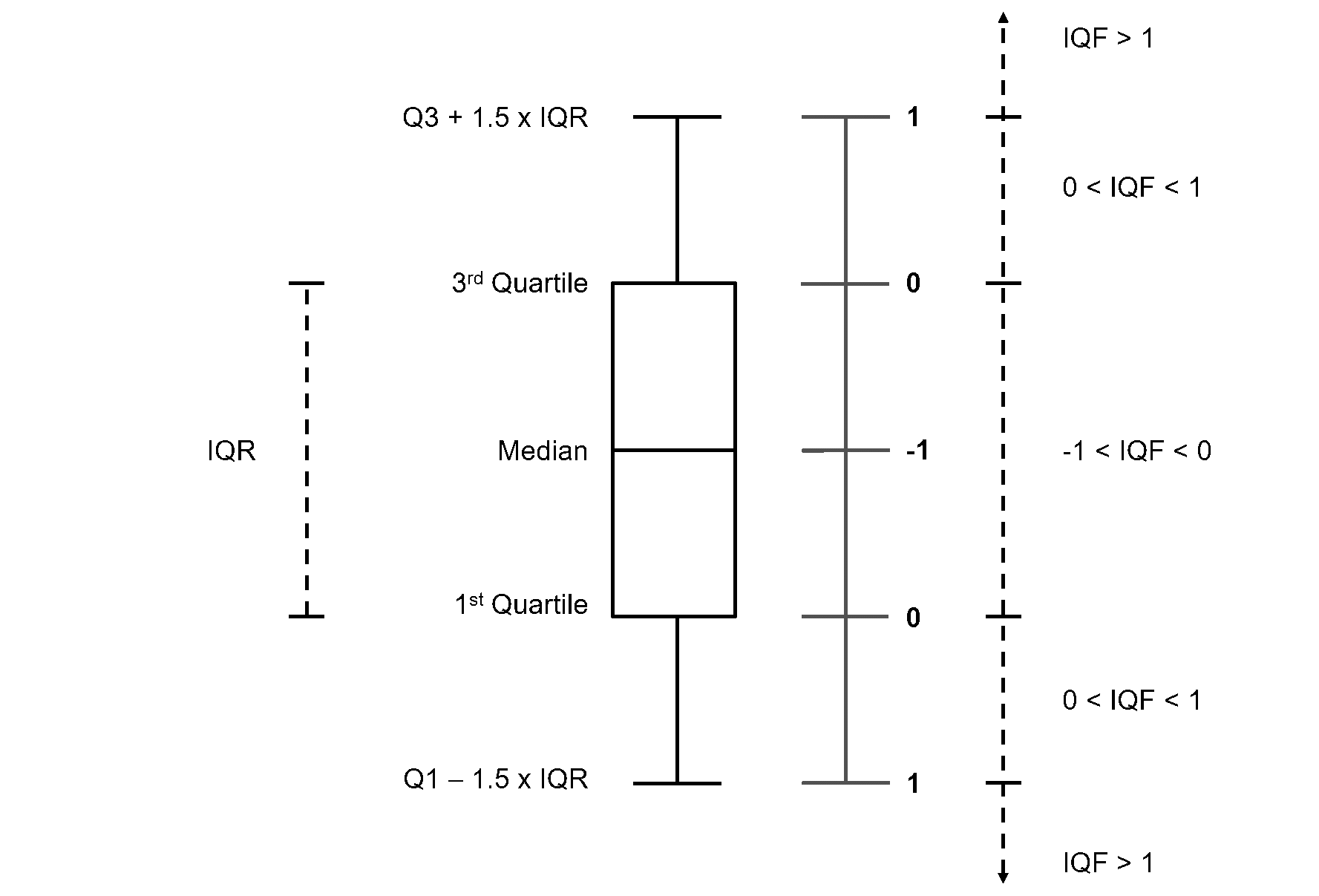


**S2 Fig. Relationship between inter-quartile factor (IQF) and the quartiles of a distribution of random permutations of x and y values.** For -1 < IQF < 0, the observed data (OD) falls between the median and the 1^st^ quartile (Q1) for values smaller than the median, or the 3^rd^ quartile (Q3) for values larger than the median. For 0 < IQF < 1, OD falls between Q1 and Q1 – 1.5 times the inter-quartile range (IQR) for values smaller than the median or Q3 and Q3 + 1.5 times IQR for values larger than the median. For IQF > 1, OD is greater than Q1 – 1.5 times IQR for values smaller than the median or Q3 + 1.5 times IQR for values larger than the median.

**References**

1. Beaufils ER. Diagnosis and recommendation integrated system (DRIS). 1973.
2. Walworth JL, Sumner ME. The diagnosis and recommendation integrated system (DRIS). Adv Soil Sci. 1987;6: 150-188.
3. Mills AJ, Milewski AV, Fey MV, Gröngröft A, Petersen M, Sirami C. Constraint on woody cover in relation to nutrient content of soils in western southern Africa. Oikos. 2013;122: 136-148.
4. Mills A, Fey M, Donaldson J, Todd S, Theron L. Soil infiltrability as a driver of plant cover and species richness in the semi-arid Karoo, South Africa. Plant Soil. 2009;320: 321-32.
5. Cade BS, Noon BR. A gentle introduction to quantile regression for ecologists. Front Ecol Environ. 2003;1: 412-420.
6. Milne AE, Ferguson RB, Lark RM. Estimating a boundary line model for a biological response by maximum likelihood. Ann Appl Biol. 2006;149: 223-234.
7. Sokal RR, Rohlf FJ. Assumptions of analysis of variance. Biometry: The principles and practice of statistics in biological research. New York: WH Freeman and Company; 1995. pp. 392-450.
8. Guo Q, Brown JH, Enquist BJ. Using constraint lines to characterize plant performance. Oikos. 1998;83: 237-245.
9. Thomson JD, Weiblen G, Thomson BA, Alfaro S, Legendre P. Untangling multiple factors in spatial distributions: lilies, gophers and rocks. Ecology. 1996;77: 1698-1715.
10. Blackburn TM, Lawton JH, Perry JN. A method of estimating the slope of upper bounds of plots of body size and abundance in natural animal assemblages. Oikos. 1992;65: 107-112.
11. Tukey JW. Exploratory data analysis. Addison-Wesley; 1977.
