# Supplementary figures and images for: Effects of anabolic and catabolic nutrients on woody plant encroachment after long-term experimental fertilization in a South African savanna
Source: PLoS One. 2017 Jun 29;12(6):e0179848. doi: 10.1371/journal.pone.0179848 (PMC5491051; doi:10.1371/journal.pone.0179848)

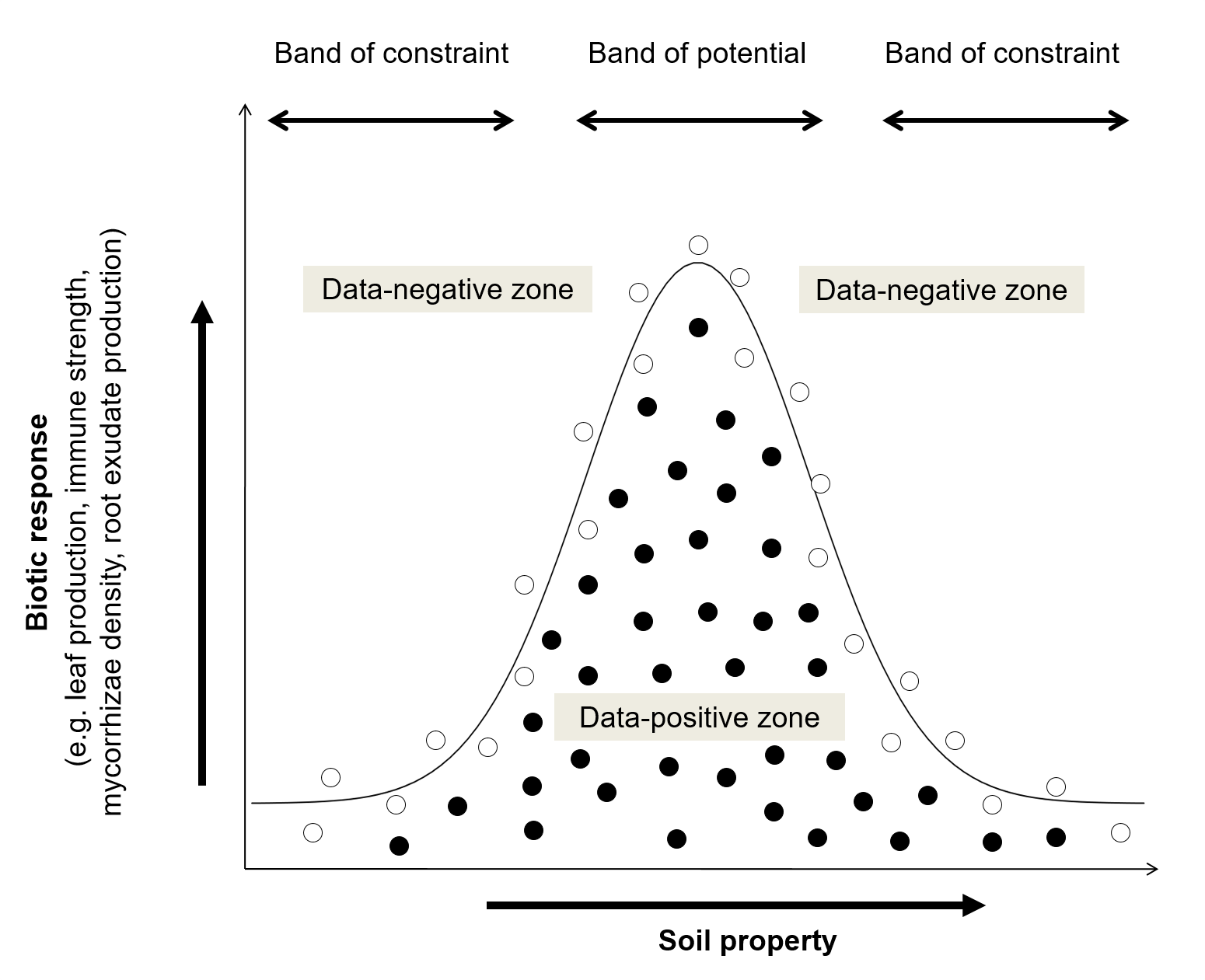

Supplement: S1 Fig — A boundary line separates a data-negative zone from a data-positive zone in a scatter plot [i, ii], which enables delineation of bands of constraint as well as potential for the y variable. [See file number 12; “S1 Fig.tif”.] (TIF) [file pone.0179848.s012.tif]

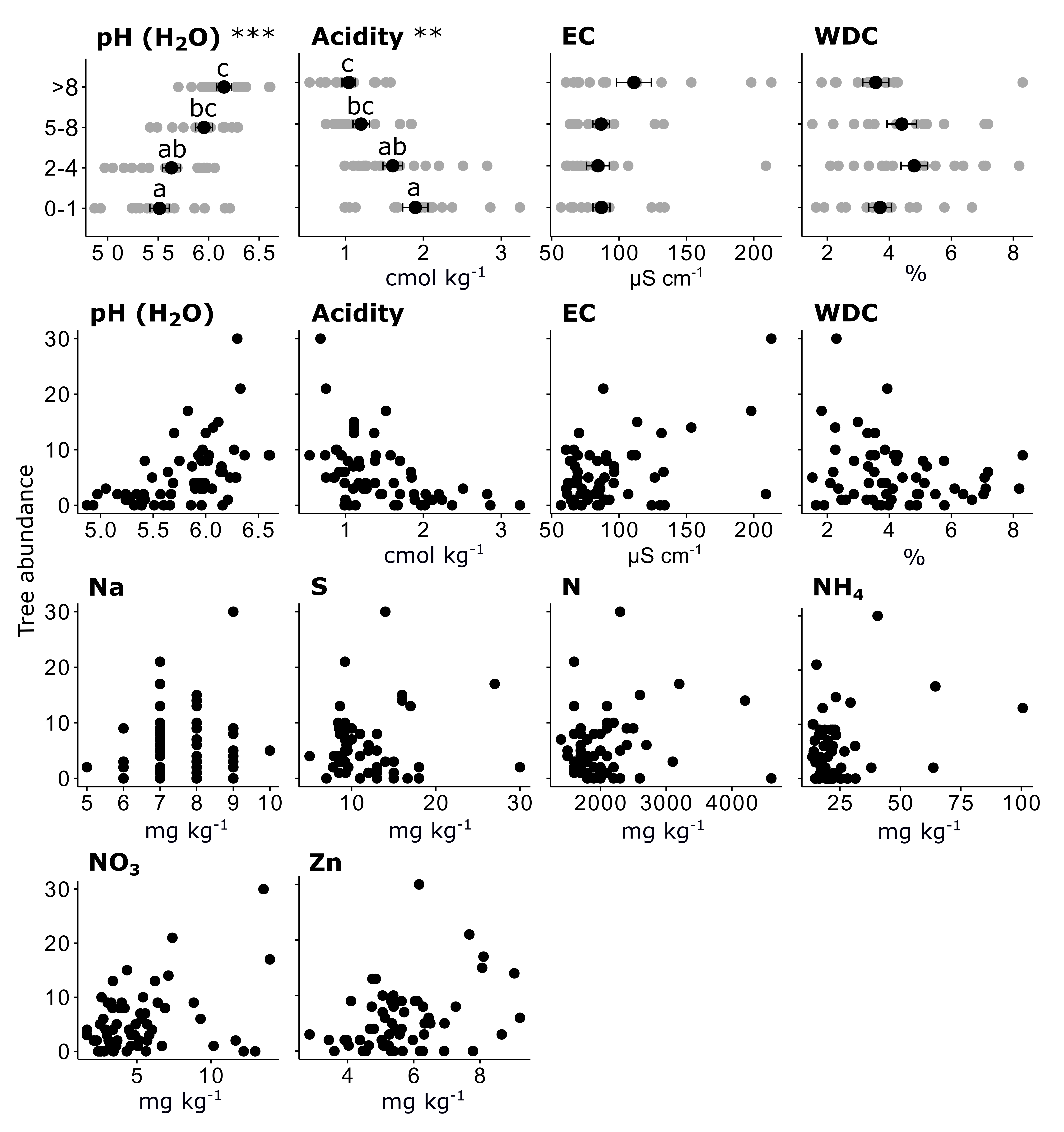

Supplement: S3 Fig — In the top row, four categories of tree abundance (i.e. 0–1, 2–4, 5–8 or >8 individual trees per plot) are shown, with data points depicted as grey circles, and means ± standard errors depicted with black circles and error bars. Asterisks show significant differences in tree abundance according to Kruskal-Wallis rank sum tests (*** p<0.0001; ** p = 0.001–0.009; * p = 0.01–0.05). Different letters designate significant differences between means (p<0.05). [See file number 14; “S3 Fig.tiff”.] (TIFF) [file pone.0179848.s014.tiff]
